# Design optimization and experimental validation of a biogas-powered stove for energy efficiency in injera baking in rural Ethiopia

**DOI:** 10.1038/s41598-025-25412-7

**Published:** 2025-11-24

**Authors:** Gabr Goshu Syum, Mulualem G. Gebrselassie, Praveen Kumar Kanti, P. C. Aruna Kumara, Mohamed Bechir Ben Hamida, H. S. Sharath Chandra

**Affiliations:** 1 Centre of Studies in Oil and Gas Engineering and Technology (CS- OGET), Eduardo Mondlane University, Av. de Moçambique, Km 1.5, Bairro Luís Cabral, Maputo, 257 Mozambique; 2 EiT-M, Mekelle University, P. O. Box 231, 7000 Mekelle, Tigray Ethiopia; 3 School of Engineering and Built Environment, Sheffield Hallam University, Sheffield, UK; 4 Department of Mechanical Engineering, Rayat Bahra Institute of Engineering and Nano Technology, Hoshiarpur, Punjab India; 5 Faculty, Department of Mechanical Engineering, Ramaiah Institute of Technology, Bengaluru, India; 6 Deanship of Scientific Research, Imam Mohammad Ibn Saud Islamic University (IMSIU), Riyadh, Saudi Arabia; 7 Department of Mechanical Engineering, NMAM Institute of Technology, Affiliated to NITTE (Deemed to be University), Karnataka, 574110 India

**Keywords:** Biogas, Injera stove, CFD simulation, Energy efficiency, Rural cooking technology, Flame uniformity, Experimental validation, Energy science and technology, Engineering

## Abstract

Injera baking is an energy-intensive daily activity in most Ethiopian households, traditionally reliant on firewood or grid electricity. Biogas presents a cleaner, more sustainable alternative, yet its application for injera baking remains limited due to excessive fuel consumption, uneven heat distribution, and inefficient stove designs. This study addresses these challenges by developing and validating a purpose-built biogas-powered injera stove. A novel burner configuration was designed, comprising five concentric feeding loops with a total of 93 carefully sized holes, and incorporating 20 mm fiberglass insulation to minimize heat loss. Unlike earlier general-purpose biogas stoves, this system was specifically engineered to meet the unique thermal and geometric requirements of injera baking. Computational Fluid Dynamics (CFD) simulations were conducted to optimize biogas flow and flame characteristics, revealing a maximum flame temperature of 1954 K with uniform distribution post-deflection by the baking pan. Experimental validation confirmed the simulation results, achieving a flame height of 33 mm and a diameter of 45 mm. The improved stove heated an 18 mm aluminum pan to 140 °C in under 10 min at a biogas consumption rate of 0.9 m^3^. The system achieved an efficiency of 43.11%, significantly higher than conventional designs as indicated in Table 3. This integrated design-simulation-validation approach demonstrates the technical feasibility of efficient, biogas-based injera baking for rural Ethiopian households.

**Supplementary Information:**

The online version contains supplementary material available at 10.1038/s41598-025-25412-7.

## Introduction

Access to reliable, affordable, and clean cooking energy remains one of the most pressing challenges in rural Ethiopia. The majority of the Ethiopian population, approximately 79% as of the latest census, resides in rural areas where traditional biomass fuels such as firewood, agricultural residues, and animal dung are the dominant sources of household energy^1^. These fuels are primarily used for cooking and baking, with injera baking being the most energy-intensive daily task^2^. Injera, a staple flatbread in Ethiopian cuisine, is traditionally baked on a circular griddle (mitad) using open fires or electric stoves. This process consumes significant amounts of energy and is typically carried out by women, exposing them to indoor air pollution, thermal stress, and long hours of fuelwood collection.

The heavy dependence on firewood has led to extensive deforestation, biodiversity loss, and declining soil fertility in many regions of Ethiopia. According to government and environmental reports, forest cover has decreased dramatically over the past few decades due to unsustainable harvesting for domestic energy use^2^. This environmental degradation contributes to broader ecological challenges, including reduced agricultural productivity, soil erosion, and declining groundwater recharge^3^. At the same time, the social and health costs associated with biomass use are substantial. Exposure to smoke from traditional stoves is a leading cause of respiratory illnesses among women and children, while the drudgery of fuel collection reduces time for education and economic activities, particularly for women and girls.

Introducing modern, clean energy solutions such as biogas is crucial for rural transformation. Biogas systems are particularly suitable for dispersed settlements where grid expansion is economically unfeasible^2^. Among the available alternatives, biogas has emerged as a promising solution due to its renewable nature, local availability of feedstock, and dual benefits of clean energy and organic fertilizer production, which is currently dominated by biomass, LPG, and kerosene^4^. Moreover, the health and climate costs of biomass emissions can be substantially mitigated through the adoption of biogas^3,5^.

Biogas is produced through anaerobic digestion of organic materials such as livestock manure, food waste, and agricultural residues, generating a combustible gas mainly composed of methane (CH_4_) and carbon dioxide (CO_2_), along with small amounts of hydrogen sulfide (H_2_S), nitrogen, and water vapor. With a typical methane content of 50–75%, biogas is well-suited for direct combustion in cooking appliances, especially when properly managed and purified^3^. Its combustion performance has been widely studied, including under homogeneous charge compression ignition (HCCI) engines using simplified reaction mechanisms^2,4^ with further insights into sensitivity to raw gas composition^2^ and the role of hydrogen sulfide content on ignition dynamics^5^.

The composition of biogas depends on the type of feedstock digested and process conditions such as temperature and the carbon-to-nitrogen (C/N) ratio. Methane is the main combustible component of biogas and is responsible for its calorific value, while other components such as CO_2_ do not contribute to energy content and are often removed or “scrubbed” during upgrading. Biogas can be purified to nearly 100% CH_4_ through distillation or other upgrading techniques. Anaerobic digestion is most efficient under neutral pH conditions (around pH 7) and can occur under two main temperature regimes: mesophilic (32–42 °C) and thermophilic (50–57 °C). The thermophilic range generally offers faster biochemical reactions due to enhanced microbial activity^2^. During anaerobic digestion, microbial communities successively break down organic matter, ultimately producing methane (CH_4_) as the final product^6^. Optimization approaches, such as artificial neural network models for landfill-based biogas systems, further highlight the potential for maximizing yields under varying process conditions^5^.

Biogas offers multiple benefits for households and communities, including clean energy generation, the production of bio-fertilizer, and improved sanitation through the more efficient use of human and animal waste^7^. It also contributes to a reduction in imported fuel oil^8^. Additionally, biogas production helps reduce the workload of women, who would otherwise spend considerable time collecting traditional biomass fuels^9^. While biogas can achieve high efficiency, up to 88%, in combined heat and power (CHP) systems, such applications require large volumes of biogas and are thus not feasible for typical household use^10^. In domestic applications, biogas efficiency varies by end-use: approximately 55% in stoves, 24% in engines, and just 3% in lamps. Studies on maximizing energy efficiency in wastewater treatment plants using waste heat recovery also offer valuable lessons for improving overall energy utilization in biogas systems^3^.

In Ethiopia, several initiatives, including the National Biogas Program of Ethiopia (NBPE), have sought to promote household-level biogas digesters, particularly in regions with high livestock populations^2^. NBPE aimed to install 15,100 domestic biogas digesters, mainly for cooking, across the country; however, only 63% of this target was achieved^10^. The uptake of biogas for injera baking has been minimal. Most existing biogas stoves are designed for general-purpose cooking and are not optimized for the specific thermal requirements and geometry of injera baking^11^. Prior attempts to develop biogas-powered injera stoves in regions such as Amhara and Oromia have often failed to gain traction due to technical shortcomings, including uneven heat distribution, excessive fuel consumption, and poor flame control^12^. These limitations have undermined user confidence and hindered the scaling of biogas technology for injera baking^13^.

This study aims to fill these gaps by designing, simulating, fabricating, and experimentally validating an improved biogas-powered injera baking stove. Using computational fluid dynamics (CFD) simulations, the research examines optimized burner configurations and pressure regulation strategies to ensure even flame propagation and efficient heat transfer. The newly developed stove features five concentric biogas loops with variable hole diameters and 20 mm fiberglass insulation to reduce heat loss and improve energy efficiency. Experimental testing assesses the system’s thermal performance, flame stability, biogas consumption, and baking effectiveness under real-world operating conditions^14^.

The novelty of this study lies in the systematic integration of computational fluid dynamics (CFD) modelling, design optimization, and experimental validation, specifically tailored for injera baking cooking practice with unique thermal and geometric requirements that have not been adequately addressed in previous biogas stove research^3,10^. Unlike earlier attempts that adapted general-purpose biogas stoves, this work develops a purpose-built design with concentric burner loops and variable hole diameters to achieve uniform heat distribution across the large baking surface^6^. The approach combines advanced simulation of flame propagation and heat transfer with practical material considerations, such as high-performance insulation, to ensure energy efficiency under real household conditions^11^. Furthermore, the study provides one of the first empirical assessments of a biogas-powered injera stove’s thermal performance, efficiency, and user-oriented effectiveness, thereby bridging the gap between laboratory concepts and community-scale application^3^. This integrated methodology represents a significant step toward scaling clean biogas solutions for culturally specific cooking practices in Ethiopia.

## Materials and methods

**Fig. 1:**
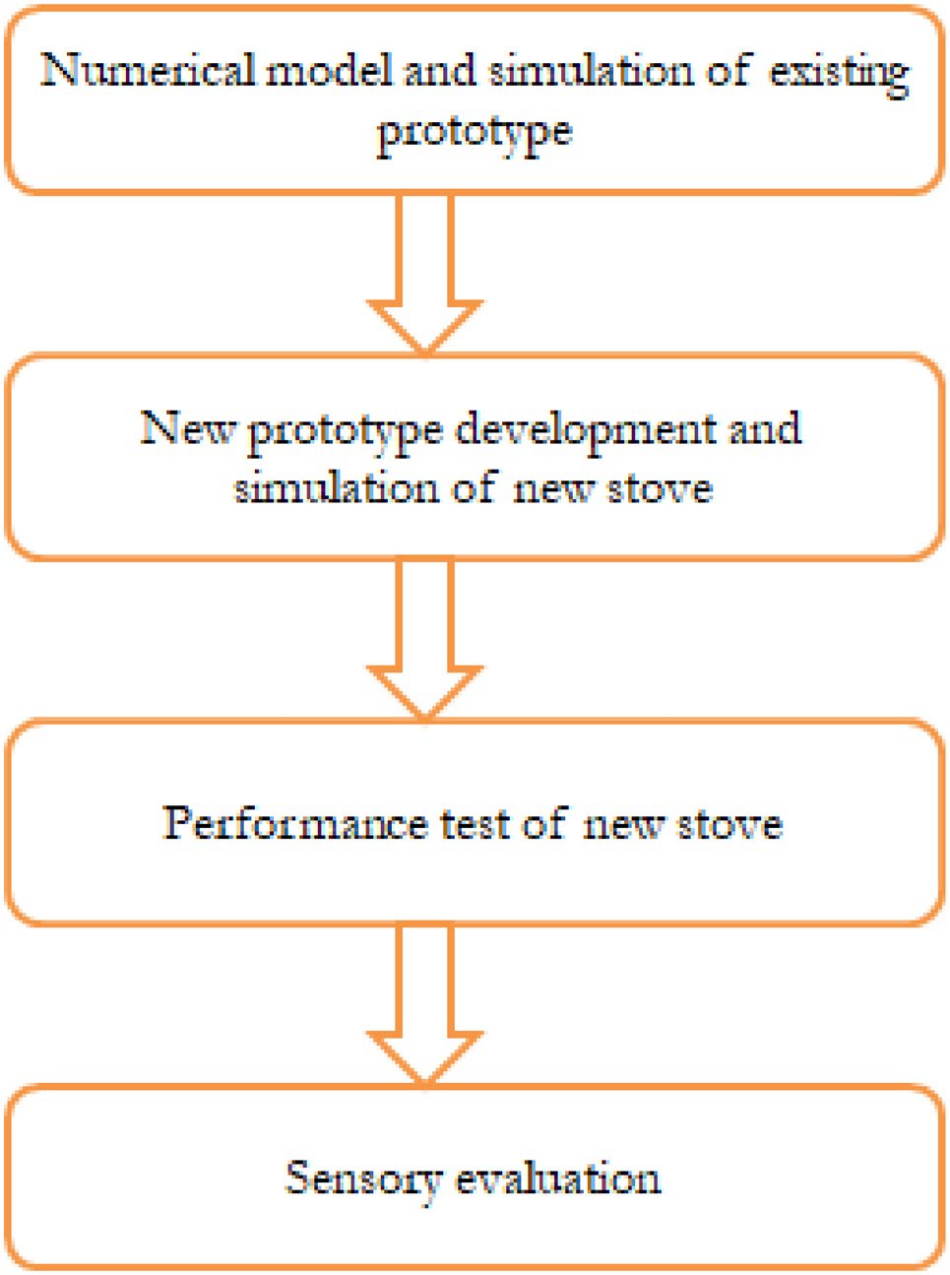
Steps of the methods.

This study used an integrated methodological approach that combined computational modeling, prototype design, experimental validation, and performance testing to develop and evaluate a biogas-powered injera baking stove. The goal was to address existing technical challenges, mainly uneven flame distribution and high biogas consumption, by improving burner design, enhancing thermal efficiency, and ensuring compatibility with household-level biogas production. The overall process included five main phases: assessing the current prototype, numerical modeling, design improvements, simulating the upgraded prototype, fabricating a physical model, and conducting experimental tests. An overview of this process is shown in Fig. 1.

### Numerical modeling of the existing stove prototype

The initial phase involved a comprehensive assessment of the performance limitations of an existing biogas stove prototype. Geometric and thermodynamic data were gathered from the system, including pipe lengths, diameters, pressure settings, the number and spacing of burner holes, and material properties in Table 1. The prototype consisted of five concentric loops with a total of 93 burner holes, all of which had equal diameters. However, biogas pressure gradually decreased along the pipe, leading to uneven flow rates and flame intensities.

**Table 1:** Data assessed for the current prototype.

Parameters	Feed pipe	Loop1	Loop2	Loop3	Loop4	Loop5
Internal diameter of pipe (m)	0.01	0.004	0.004	0.004	0.004	0.004
Internal diameter of the gate valve (m)		0.003	0.003	0.003	0.003	0.003
Length of pipe (m)	3.5	1.57	1.256	0.942	0.628	0.314
Pressure set (Pa)	1.25					
Number of holes		32	26	19	12	4
Gap between hole (m)		0.005	0.005	0.005	0.005	0.005
Gap between Loops (m)		0.005	0.005	0.005	0.005	0.005
The viscosity of biogas ($\text{Pas})$	0.000011					
Density	1.1					
Material	Aluminum					

To investigate these flow behaviors, a computational fluid dynamics (CFD) model was developed using ANSYS Fluent 15. The geometry was reconstructed in 3D using CATIA and meshed using a structured mesh to ensure accuracy. Boundary conditions included inlet pressure (1.25 bar)^15^, flow velocity, and material properties of biogas. A laminar flow model was used, given the low Reynolds number characteristic of the biogas flow^16^. The objective of this simulation was to understand velocity gradients, identify pressure losses, and evaluate the overall flow distribution through the concentric loops^11^. From the known value of the pressure and volume flow rate through the holes, it needs to be the same for equal distribution of heat through the baking pan, P = 25,000 Pa. Hence, in the existing prototype, there are 93 holes, so the pressure must be distributed equally to get the same volume flow rate through the holes.

$$P1 = P2 = P3 = \ldots . = P93 = P\;{\text{and}}\;Pt = 93*P\;{\text{and}}\;P = \frac{Pt}{{93}}$$

**Fig. 2:**
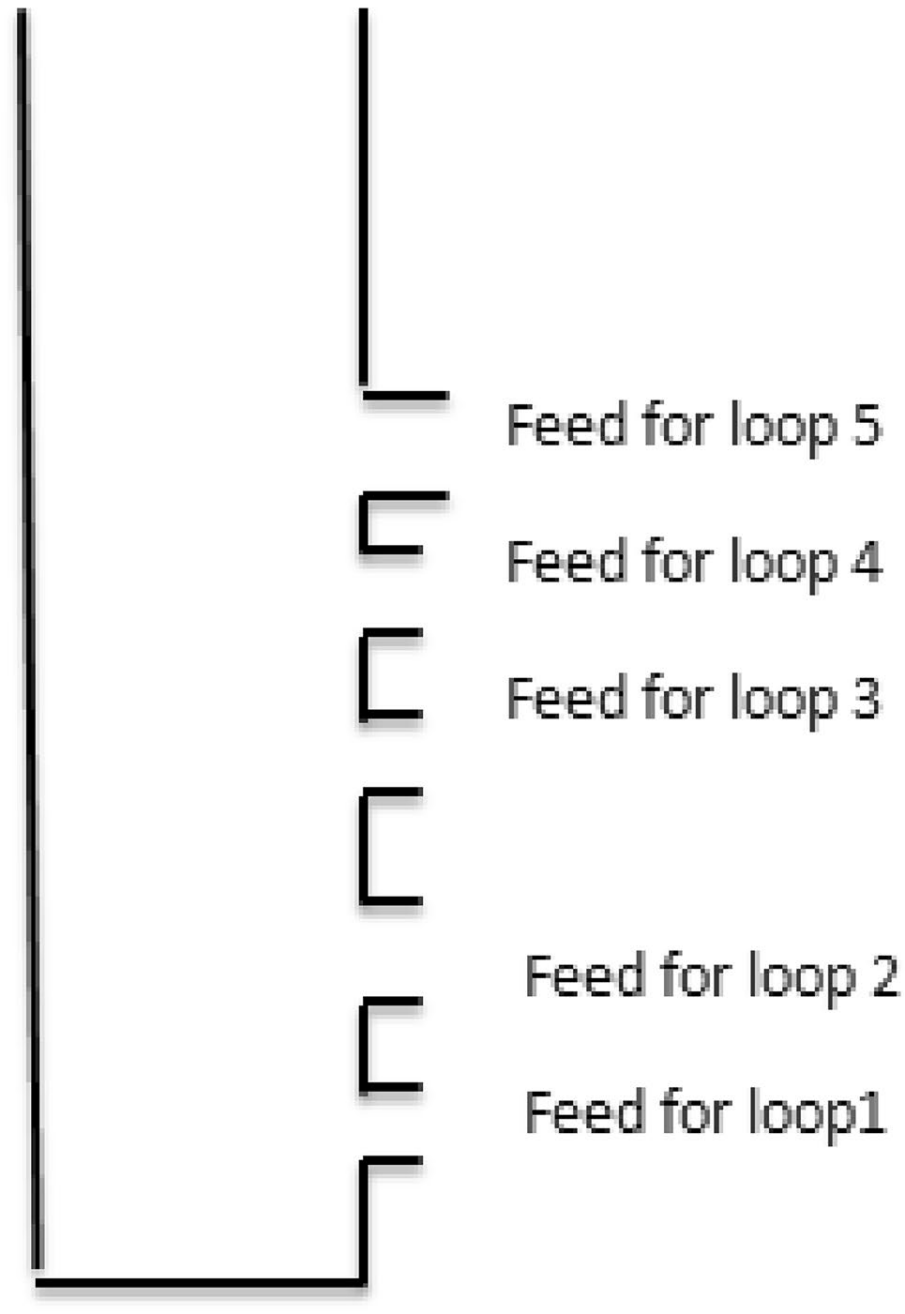
Biogas stove distributor.

As illustrated in Fig. 2 (biogas stove distributor**)** below, the system comprises five feeding loops, each supplying gas to a distinct number of burner holes. These loops feed a different number of holes.

- Loop 5 feeds 32 holes
- Loop 4 feeds 26 holes
- Loop 3 feeds 19 holes
- Loop 2 feeds 12 holes
- Loop 1 feeds 4 holes

The pressure required for each loop was determined as follows.

Pressure for loop 5

$$P5 = 32*P$$

Similarly, for loop 4

$$P4 = 26*P$$

Pressure for loop 3

$$P3 = 19*P$$

Pressure for loo2

$$P2 = 12*P$$

Finally, pressure for loop 1

$$P1 = 4*P$$

The total pressure is the summation of loop pressures.

$$Pt = P1 + P2 + P3 + P4 + P5$$

Biogas flow into the feeding loop can be represented by mass flow rate as follows.

Mass flow rate for loop 1

$$\dot{Q}_{1} = \frac{{P_{1} *\pi * r_{pipe}^{4} }}{8*\mu *l}$$

$$m = \rho_{biogas} * \dot{Q}$$

For loop 2

$$\dot{Q}_{2} = \frac{{P_{2} *\pi * r_{pipe}^{4} }}{8*\mu *l}$$

For loop 3

$$\dot{Q}_{3} = \frac{{P_{3} *\pi * r_{pipe}^{4} }}{8*\mu *l}$$

For loop 4

$$\dot{Q}_{4} = \frac{{P_{4} *\pi * r_{pipe}^{4} }}{8*\mu *l}$$

Finally, for loop 5.

$$\dot{Q}_{5} = \frac{{P_{5} *\pi * r_{pipe}^{4} }}{8*\mu *l}$$

*Since* $\mu$—Viscosity of biogas. *r*—Radius of the gate valve. *l*—Length from source to distributor. $\dot{m}$—Mass flow rate.

### Simulation of the existing biogas prototype

**Fig. 3:**
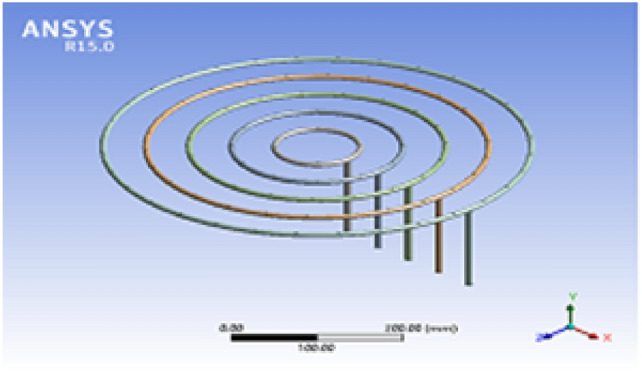
Biogas stove geometry.

**Fig. 4:**
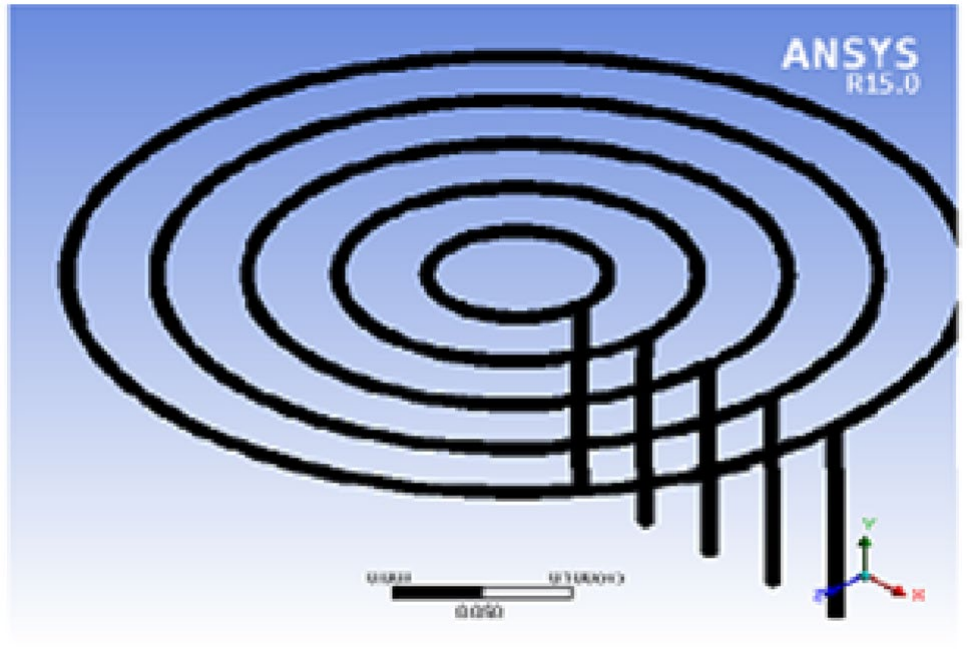
Biogas stove mesh in ANSYS.

The simulation is conducted in ANSYS Fluent 15 by using numerical modeling values as boundary conditions. The software is used for simulating the motion of biogas in the feeding loops and for flame characteristics. It is simulated at the pressure-driven flow, and the model is laminar, simulating the motion of biogas at feeding loops 1, 2, 3, 4, and 5. The boundary condition for the inlet was = 0.000061 kg/s, 0.000183 kg/s, 0.00028 kg/s, = 0.00039 kg/sand 0.00048 kg/respectively. In below Fig. 3 shown for biogas stove geometry, and Fig. 4 indicates for biogas stove mesh in ANSYS Workbench.

### Problem identification of the existing prototype and improvement

The problem in the existing system was a lack of equal volume flow rate at each hole. This happened due to velocity (pressure) reduction across the pipe length, and the holes are equal in diameter. There was no way to compensate for the reduction. This creates uneven flame characteristics and propagation, such as length and width in holes. As a result, it creates uneven heat distribution across the baking pan. Operating pressure (0.25 bar) required a higher amount of energy for baking injera. The improved design introduced progressively increasing hole diameters along each feeding loop to equalize the biogas volume flow rate despite pressure reductions. Additionally, the new configuration minimized sharp turns and reduced pipe lengths to lower pressure drop. The total number of holes remained 93, distributed across five feeding loops, but hole sizing was customized for each loop segment to optimize flame uniformity.

### Simulation of the improved biogas stove design

**Fig. 5:**
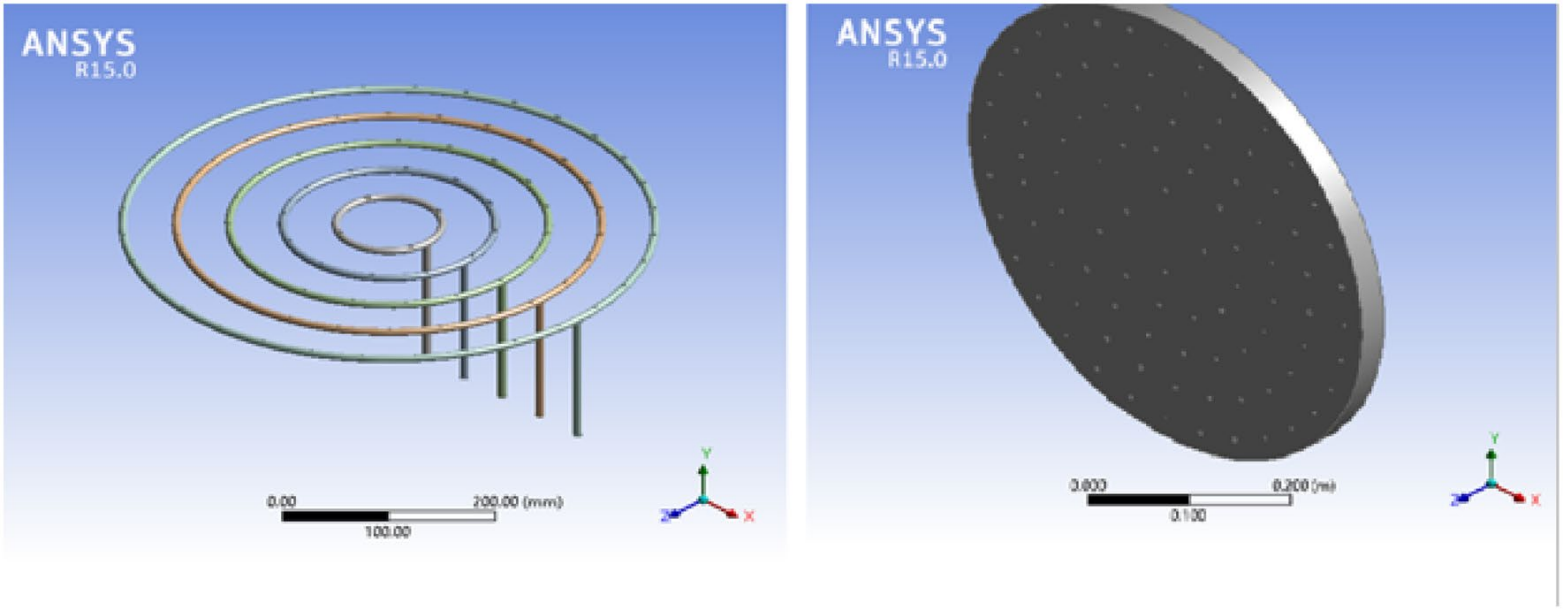
Geometry for biogas stove.

**Fig. 6:**
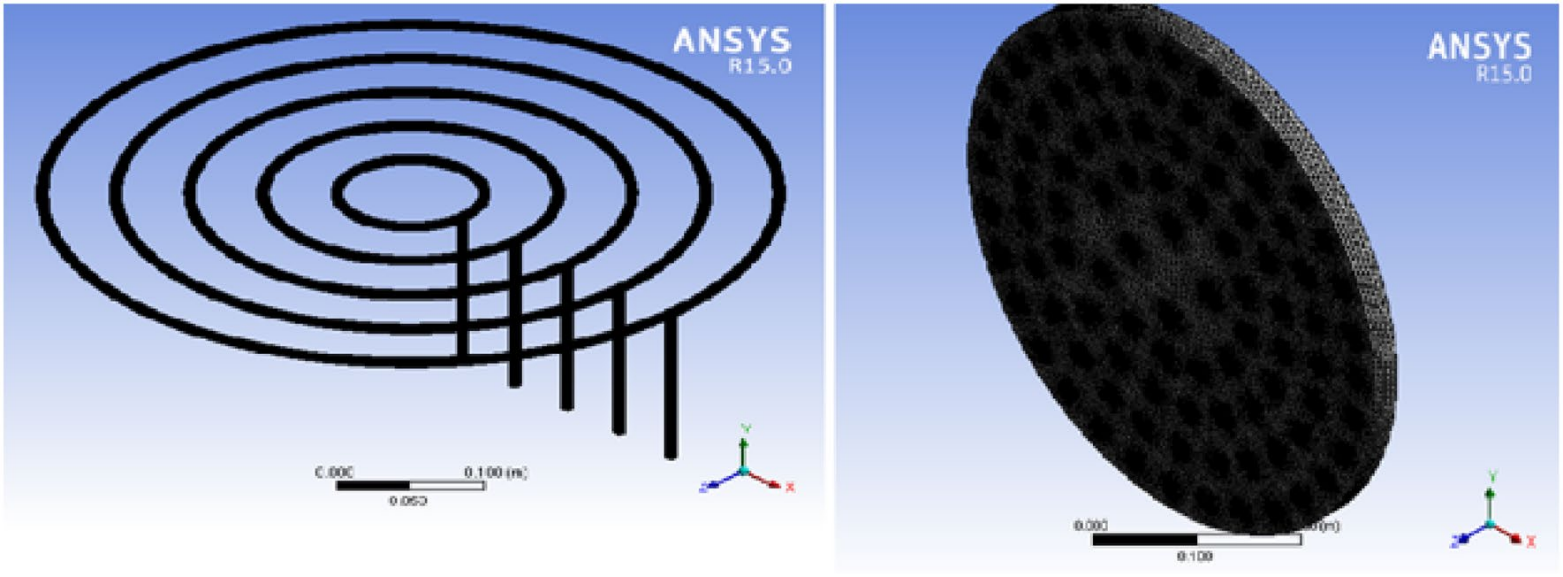
Biogas mesh in ANSYS Workbench.

An Ansys simulation is conducted on the new prototype. The simulation is done by drawing the 3D Geometry, which represents the new stove and discretizing the geometry to the best mesh size manually as shown below in Figs. 5 and 6.

**Fig. 7:**
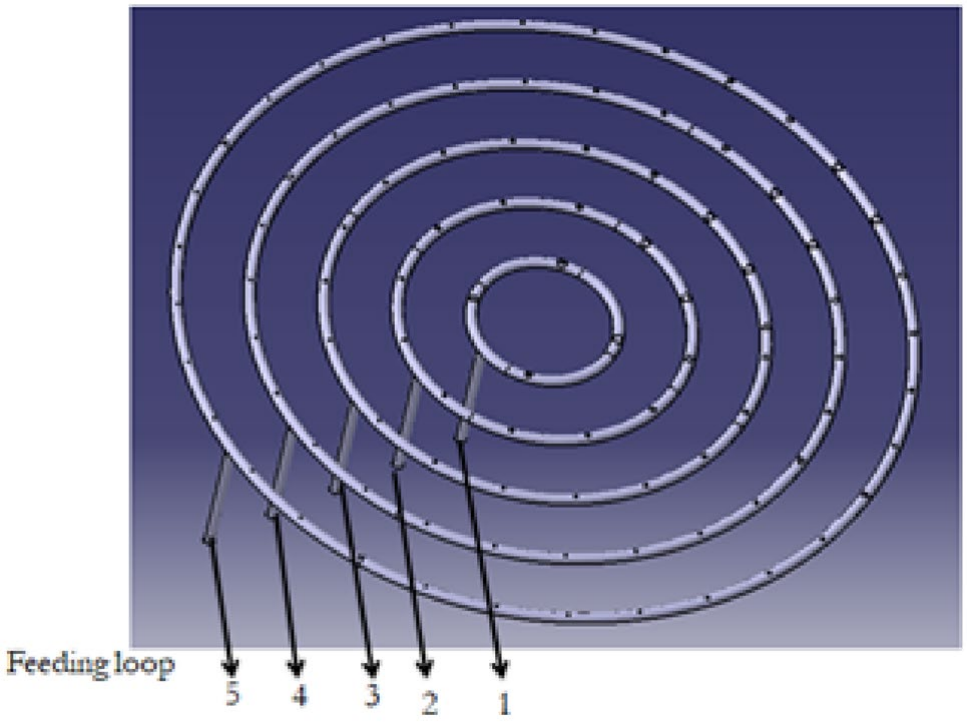
Shown us improved stove geometry drawn in CATIA.

**Fig. 8:**
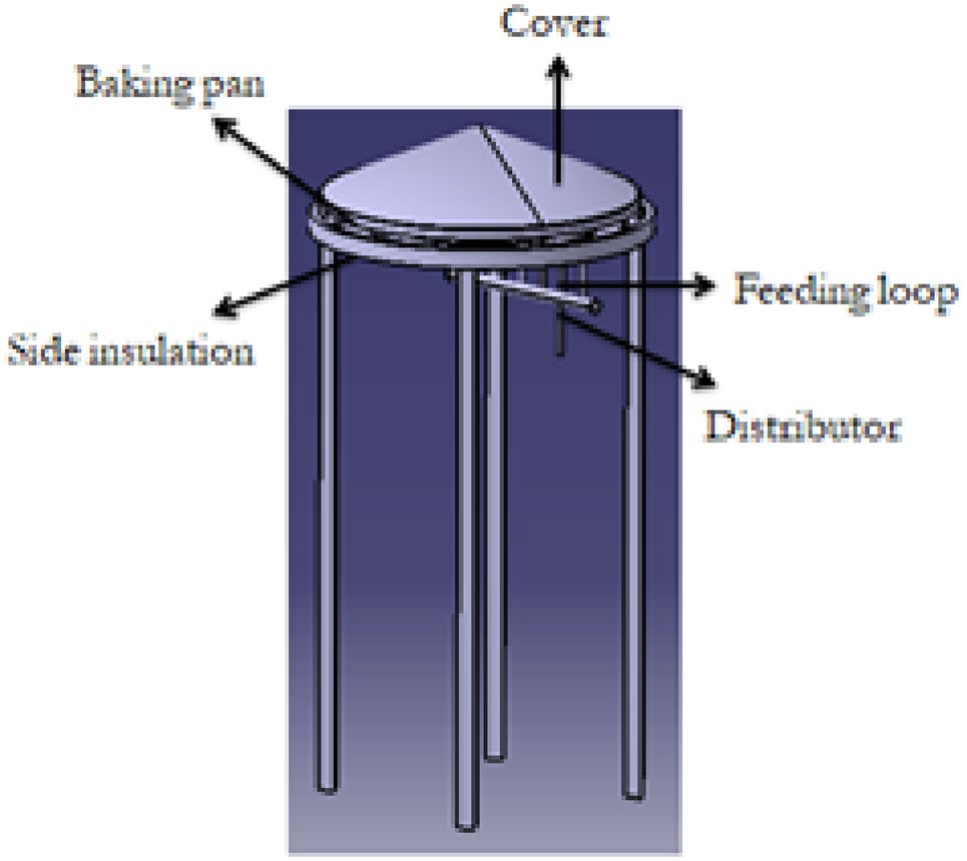
Illustrates for burner.

**Fig. 9:**
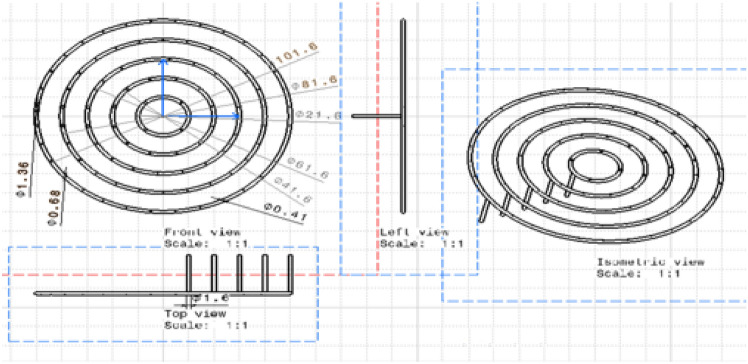
Describes for full stove with drafting of the burner.

The improved stove geometry was recreated in CATIA, including five concentric biogas loops with different hole diameters and strategic layout adjustments (Figs. 7, 8, and 9). The flow behaviour of biogas through the redesigned burner was simulated using CFD in ANSYS Fluent 15.

The analysis focused on:

- *Mass and momentum conservation* using cylindrical coordinate formulations.
- *Laminar flow* regime under pressure-driven boundary conditions.
- *Non-premixed combustion* modelling using species transport equations.
- *Radiative heat transfer* using the discrete ordinates (DO) radiation model.

*Conservation of mass* Conservation of mass in the cylindrical coordinate system^17^ is given by the following equation:

$$\frac{\partial \rho }{{\partial t}} + \frac{1}{r}\frac{{\partial \left( {\rho rVr} \right)}}{\partial r} + \frac{{\partial \left( {\rho V\theta } \right)}}{\partial \theta } + \frac{{\partial \left( {\rho Vz} \right)}}{\partial z} = 0$$

The system is one-dimensional steady fluid flow, so the continuity equation will reduce to the following for a steady-state

$$\frac{\partial \rho }{{\partial t}} + \frac{1}{r}\frac{{\partial \left( {\rho rVr} \right)}}{\partial r} + \frac{{\partial \left( {\rho V\theta } \right)}}{\partial \theta } + \frac{{\partial \left( {\rho Vz} \right)}}{\partial z} = 0$$

*Conservation of momentum* Linear momentum conservation in cylindrical coordinates, given different directions and with the following assumptions, can be simplified, steady state, no change in velocity in the ϴ and r (V $\theta = Vr = 0)$ and no gravitational force.

Boundary conditions were derived from volumetric flow rate calculations based on the thermal energy requirement for injera baking (3.3 kW), calorific value of biogas (22 MJ/m^3^), and desired uniform temperature distribution across the baking pan. The flow rate for each loop was set proportionally to the number of holes it served. A total system pressure of 2148 Pa was determined as the optimal operating condition for achieving uniform flame propagation.

Simulations were conducted for both pre-deflection (direct flame jets) and post-deflection (flames interacting with the underside of the pan) conditions to assess heat distribution and flame geometry. Flame shape, temperature contours, and velocity fields were analysed to ensure that the improved configuration provided uniform heating.

### Thermal modeling and heat loss analysis

Thermal analysis of the system was performed to estimate heat losses through convection and radiation. Heat transfer from the aluminium baking surface, sidewalls, and bottom of the stove was assessed using standard thermodynamic equations and Rayleigh number correlations for natural convection. The addition of 20 mm fiberglass insulation to the stove walls was designed to minimize heat losses, enhance thermal efficiency, and improve user safety.

$$R_{a} = \frac{{\left( { gcos\theta *\beta *\left( {T_{s} - T_{\infty } } \right)*L^{3} } \right)}}{{\nu^{2} }}*P_{r}$$

The convective heat loss from the aluminium pan cover, inclined at an angle, was determined using slant height and surface geometry. Heat loss through the bottom and side surfaces was also measured using the thermophysical properties of insulation and stove materials. These values were then used in energy balance calculations and to estimate the overall system efficiency.

### Fuel combustion model

To evaluate the combustion performance of the biogas-powered injera stove, a non-premixed combustion model was implemented within the CFD framework. The model formulation, assumptions, and validation steps are detailed below.

#### Model formulation

The combustion of biogas was modelled using the species transport approach, which solves mass conservation equations for each chemical species involved in the reaction. The governing transport equation for species III is expressed as:

$$\frac{{\partial \left( {\rho Y_{i} } \right)}}{\partial t} + \nabla \cdot \left( {\rho {\mathbf{u}}Y_{i} } \right) = - \nabla \cdot {\mathbf{J}}_{i} + R_{i}$$

where *ρ* = density of the mixture (kg/m^3^). Yi = mass fraction of species iii. u = velocity vector (m/s). Ji = diffusive flux of species I (kg/m^2^ s). Ri = net rate of production/consumption due to chemical reactions (kg/m^3^ s)

The eddy-dissipation concept (EDC) was employed to couple turbulence and chemical kinetics. The single-step reaction approximated the global oxidation of methane:

$${\text{CH}}_{4} + 2{\text{O}}_{2} \to {\text{CO}}_{2} + 2{\text{H}}_{2} {\text{O}}$$

To account for the presence of CO_2_ and H_2_S in biogas, their dilution and inhibition effects on flame temperature and reaction rates were incorporated based on simplified reaction mechanisms proposed for HCCI engine simulations ^2,4,5^.

#### Assumptions

Several assumptions were made to simplify the combustion model:

1. A laminar combustion regime was assumed locally due to the low Reynolds number of the biogas flow through the burner holes.
2. Steady-state operation was considered, ignoring transient ignition and extinction phases.
3. Biogas composition was represented as 60% CH_4_, 38% CO_2_, and 2% H_2_S, reflecting average digester output in Ethiopia.
4. Radiation heat transfer was modelled using the Discrete Ordinates (DO) model, with absorption/emission coefficients assigned to major participating gases (CO_2_ and H_2_O).
5. Secondary air entrainment occurred naturally around burner jets and was not forced.
6. Thermophysical properties (density, viscosity, specific heat) were assumed to be constant at operating temperature ranges.

#### Model validation

The combustion model was validated by comparing simulated results with experimental flame observations and published biogas combustion studies:

- The maximum simulated flame temperature of 1954 K closely matched measured flame temperatures, with less than 5% deviation.
- Flame dimensions (height ~ 33 mm, width ~ 45 mm) predicted by CFD agreed with experimental recordings, confirming the accuracy of species transport and energy balance formulations.
- Results are consistent with values reported in prior experimental work on biogas combustion^8,9^ and with computational studies on simplified reaction mechanisms for biogas in HCCI systems^2,3^. This agreement between simulated and experimental data provides confidence that the combustion model reliably captures the main thermo-fluid and chemical phenomena in the improved stove, enabling its use as a predictive tool for design optimization^4^.

### Prototype fabrication

**Fig. 10:**
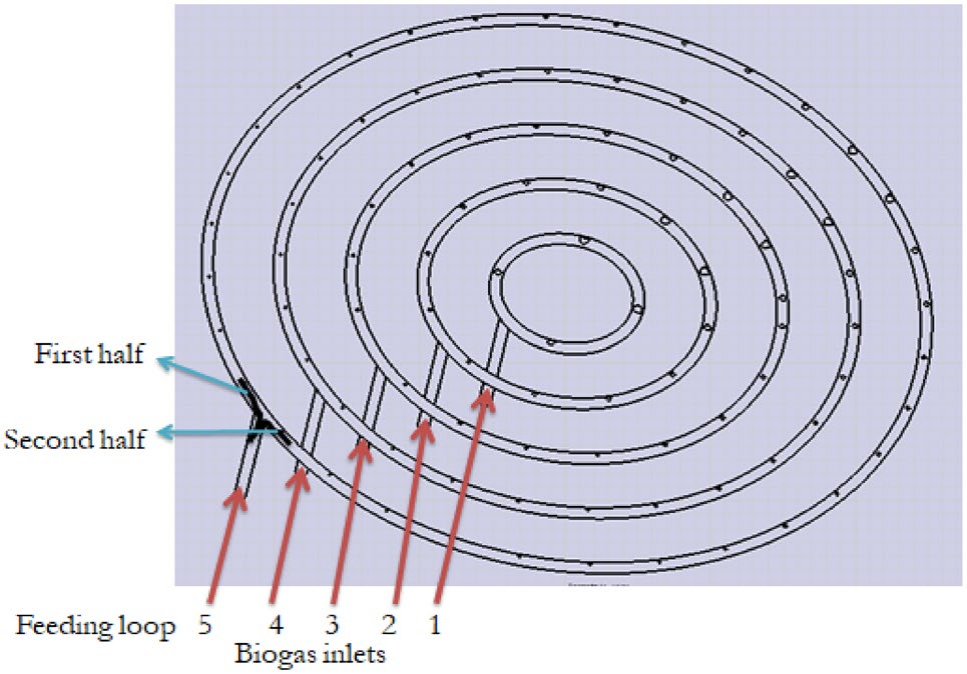
Biogas feeding loop of the stove.

**Fig. 11:**
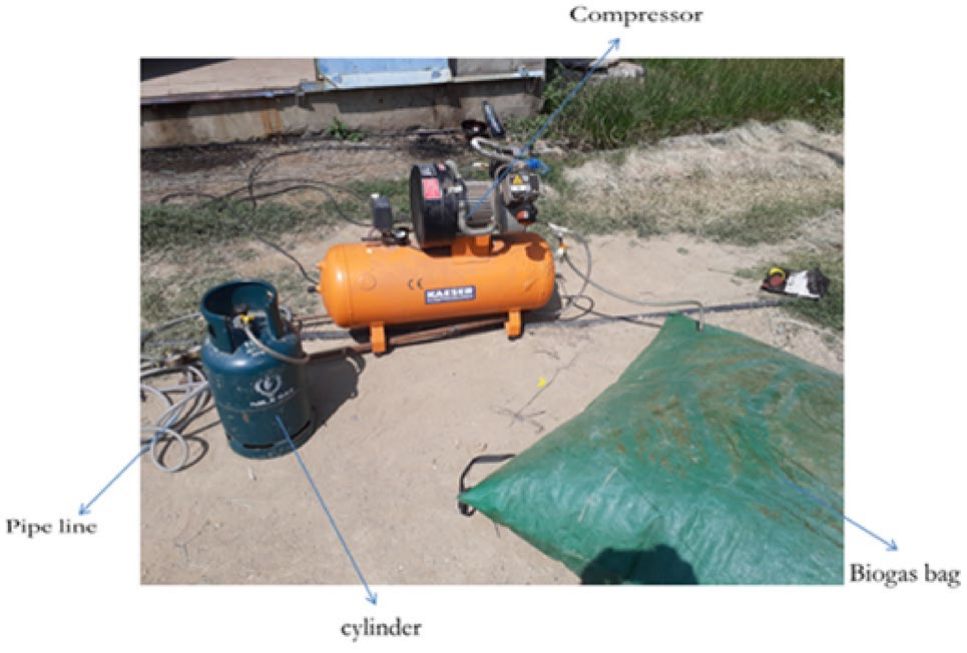
Implies setup for flame propagation and characteristics.

The improved stove was physically manufactured based on the final CAD design and simulation results. The fabrication process was carried out at Badru Muleta PLC in collaboration with the workshop facilities of Mekelle University. The following steps were taken:

- Burner loops and feed pipes were made from aluminium and copper using arc and oxy-acetylene welding as shown in Fig. 10.
- Hole drilling was performed using radial drilling machines, with precise variation in diameters as determined from simulation results.
- Insulation was applied around the burner walls using fiberglass sheets to minimize convective and radiative losses.
- A biogas storage system was integrated using high-pressure cylinders (12 bar), filled from a digester via a compression and packaging unit. The flame was created by burning biogas in each hole with an adequate air supply. The propagation and characteristics testing propagation and characteristics of the flame depend on the flow rate of biogas. Biogas is compressed and stored in a cylinder at 12 bars as shown in Fig. 11.

**Fig. 12:**
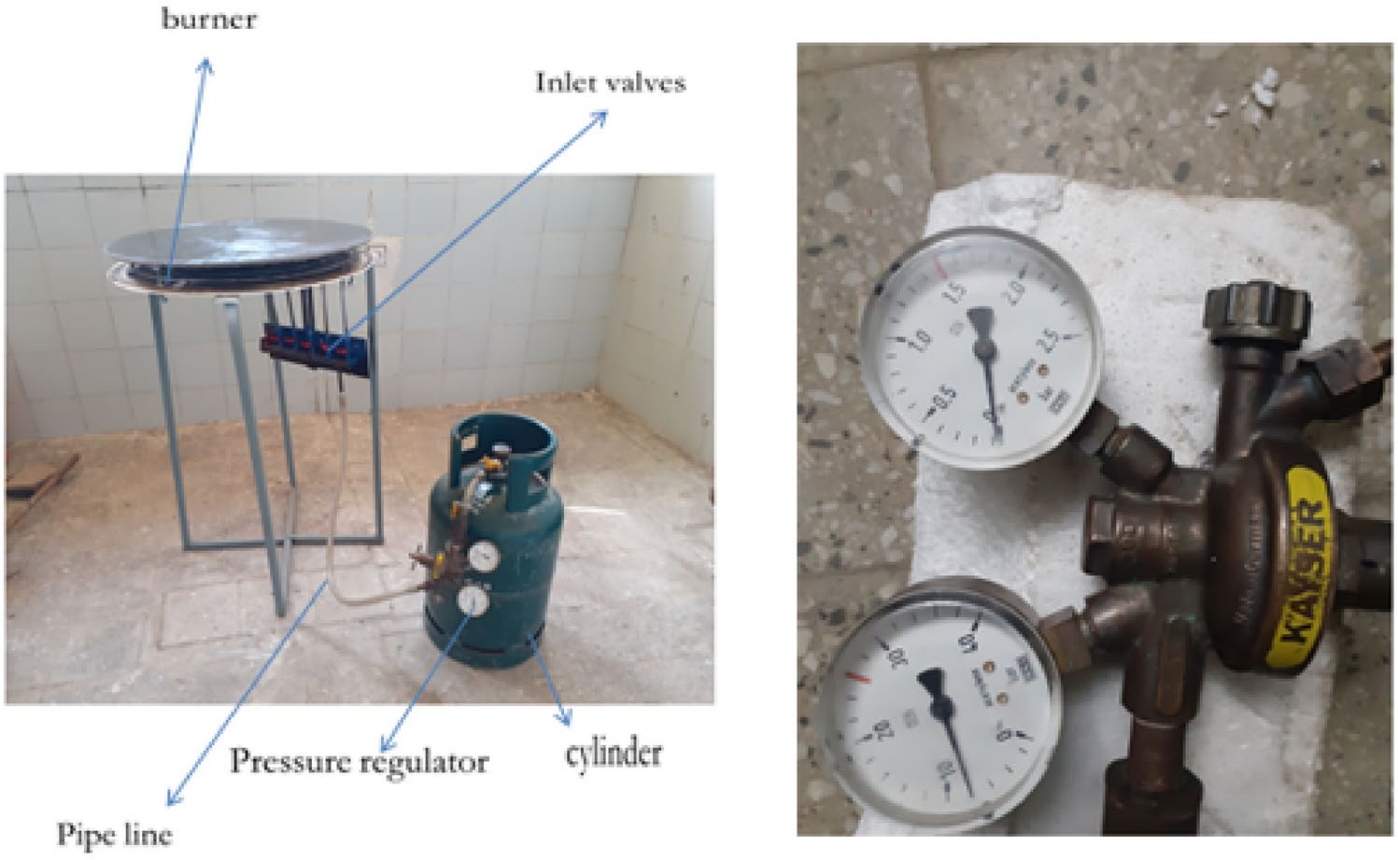
Setup of the biogas stove system showing the main components, burner, inlet valves, cylinder, pressure regulator, and pipeline (left, and a close up view of the pressure regulator with dual gauges for monitoring gas flow and pressure(right).

This stored biogas is fed into the biogas burner at a total pressure of 2152 Pa, which is divided into feeding loops 1,2,3,4, and 5 with 4 Pa, 66 Pa, 250 Pa, 634 Pa, and 1198 Pa, respectively. The test is recorded by using a Samsung Galaxy A10 selfie camera, and the setup is shown below Figs. 11 and 12.

**Fig. 13:**
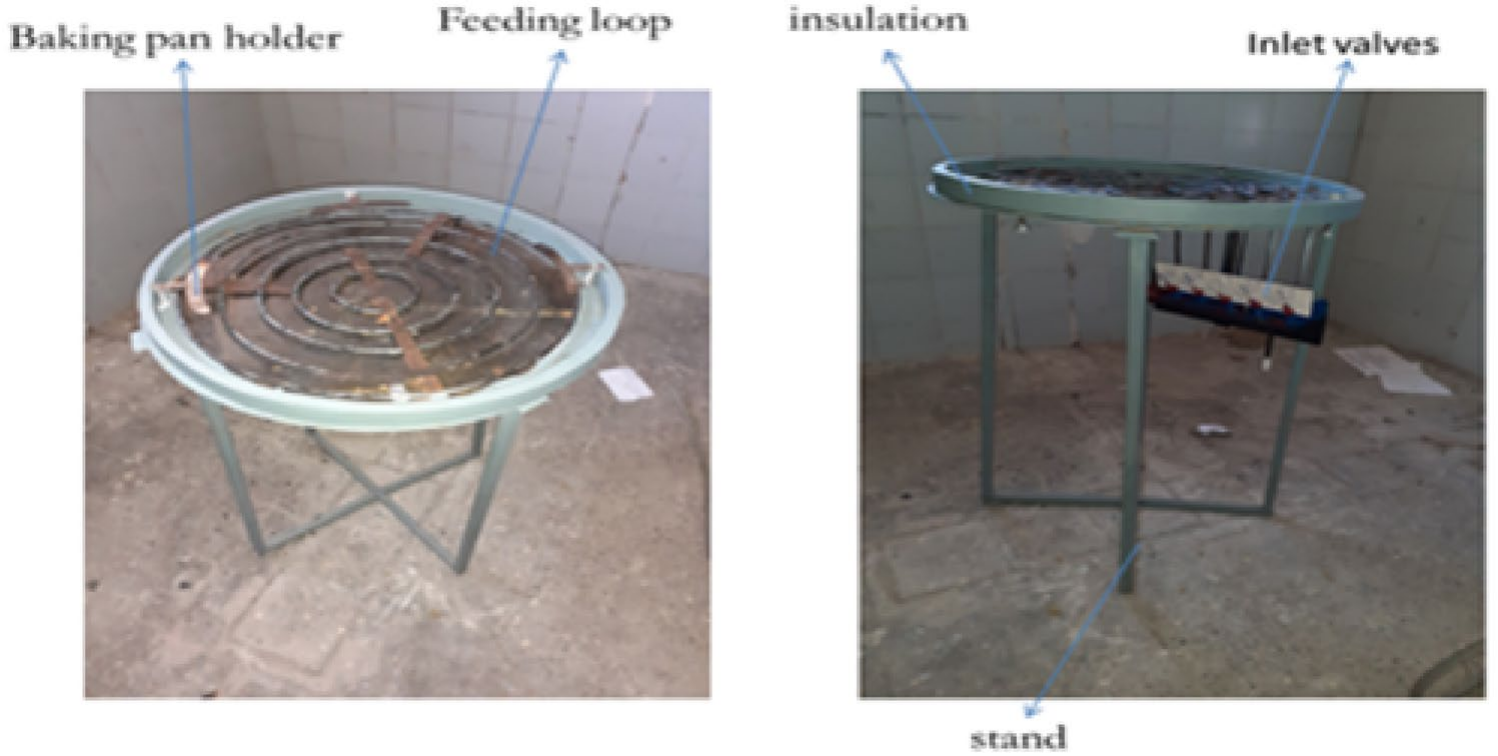
Shows for improved stand stove prototype manufactured.

**Fig. 14:**
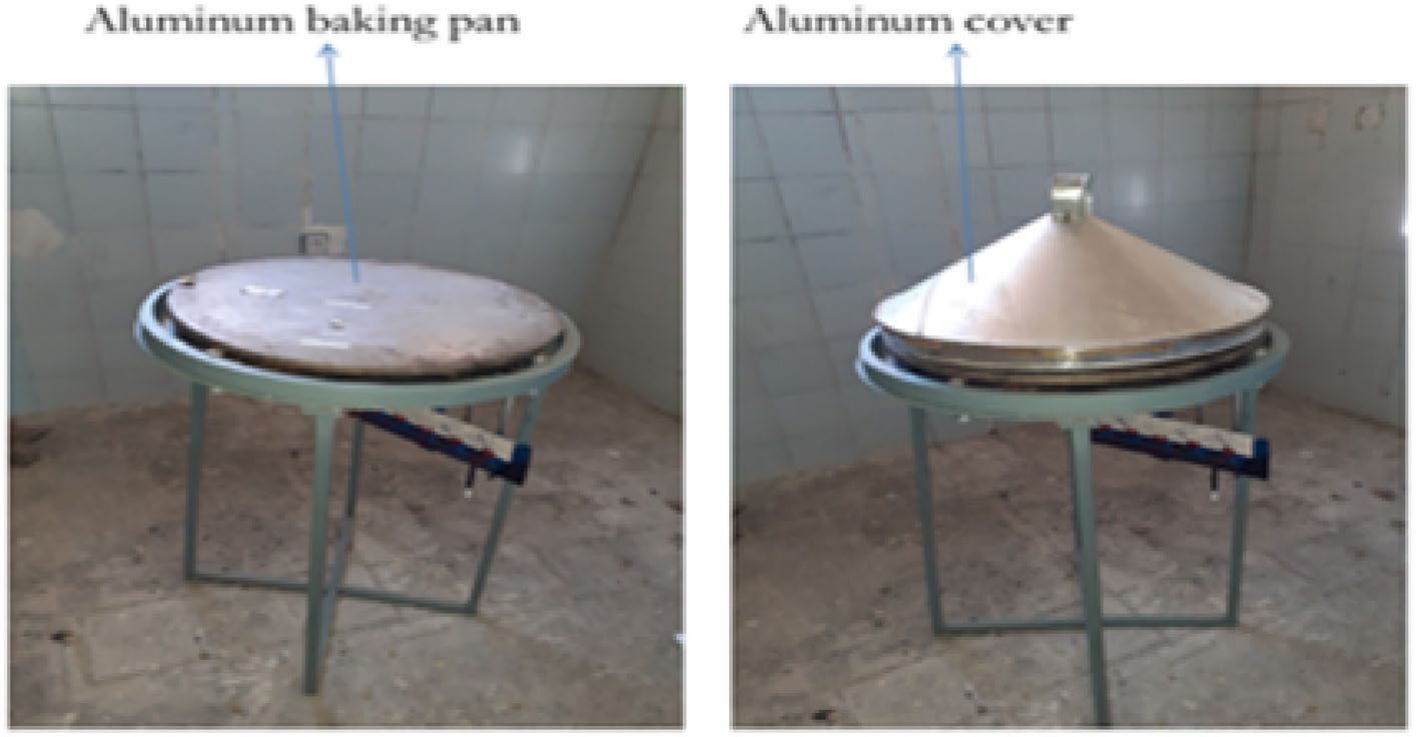
Indicated for improved aluminum baking pan and cover stove prototype manufactured.

The final assembly consisted of the burner, stand, support structure, pressure regulator, and a pan mounting system, as shown below in Figs. 13 and 14.

### Experimental setup and testing procedures

The experimental phase included three main tests: flame characterization, thermal distribution measurement, and injera baking trials.

#### Flame testing

Biogas was supplied to the stove at a pressure of 2152 Pa, with loop-wise division as per the simulation setup. Flame shape, height, and width were observed visually and recorded with a digital camera. Measurements were taken after the flame stabilized under steady-state conditions. Flame colour served as an indirect indicator of combustion completeness. It was the major loss that occurred from the flame as far as the flame is open to the environment for the sake of secondary aeration. It is important to notice that the adiabatic flame temperature of the combustion of biogas varies with distance over the burner^18^. Thus, overall heat losses would be the summation of heat loss through convection and radiation and can be computed as follows:

$$Q_{Conv} = h_{free\;air} *A_{air\;inlet} *\left( {T_{flame} - T_{ambi} } \right)$$

#### Thermal performance testing

To evaluate heat distribution, the baking pan was divided into four quadrants. K-type thermocouples were placed at multiple points (centre, edges, quarter lines) on the pan surface. Temperature readings were recorded over time as the stove operated continuously, enabling an assessment of heating uniformity and thermal response time. Cooling behaviour was also observed to understand heat retention after shutdown. The thermal efficiency is given as follows.

$$\eta_{Thermal } = \frac{used\;energy}{{input\;energy}}$$

#### Injera baking trials

**Fig. 15:**
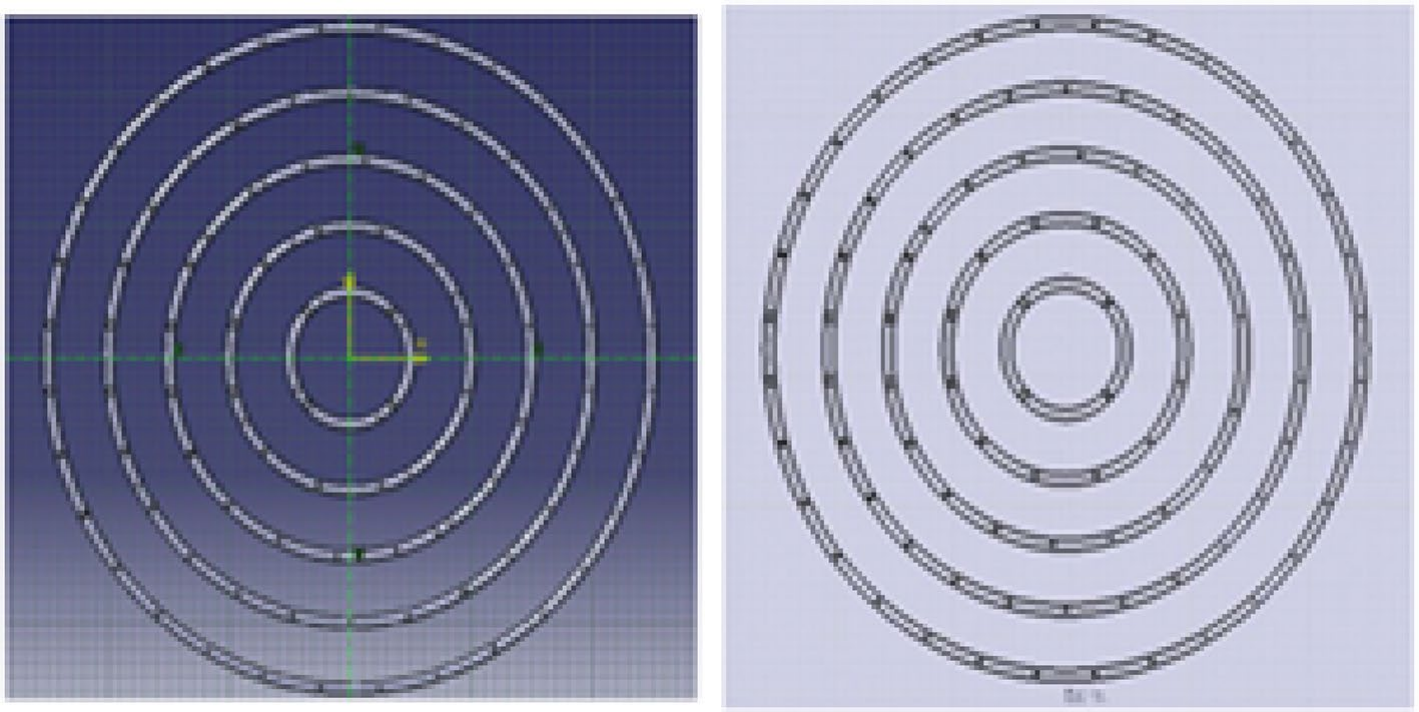
Top view of the biogas feeding loop.

**Fig. 16:**
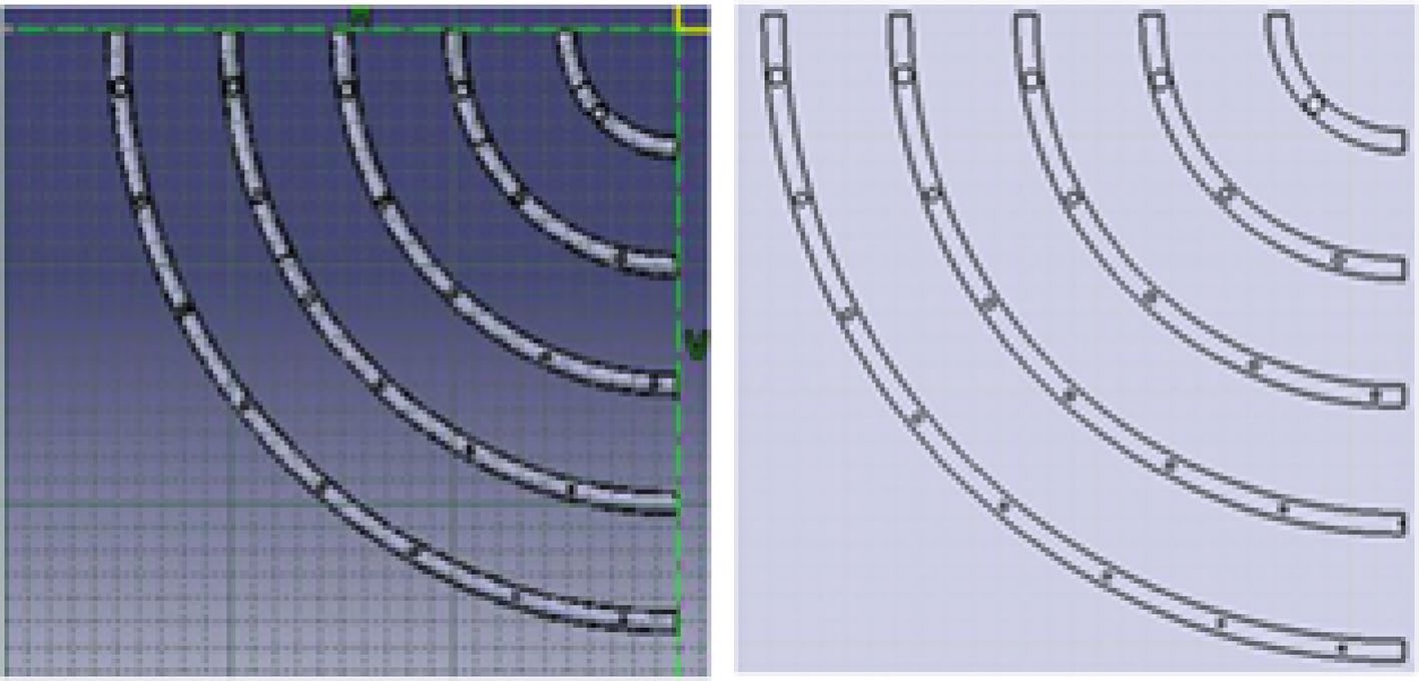
Illustrates for quarter of the biogas feeding loop.

Once the manufacturing process and the prototype development were completed, the experimental setup for performance testing of the system was fixed as shown in Fig. 15. Thus, the performance of the system was tested by checking equal temperature distribution across the baking plate. To do this testing, first the baking plate was equally divided into quarters as shown in Fig. 16 below so that the K-type thermocouples were adjusted in six places according to the distance of the flame ports for both x and y-axis of the baking plate partition. Biogas is compressed and stored in a cylinder at 12 bars. This stored biogas is fed into the biogas burner at a total pressure of 2152 Pa, which is divided into feeding loops 1,2,3,4, and 5 with 4 Pa, 66 Pa, 250 Pa, 634 Pa, and 1198 Pa, respectively.

**Fig. 17:**
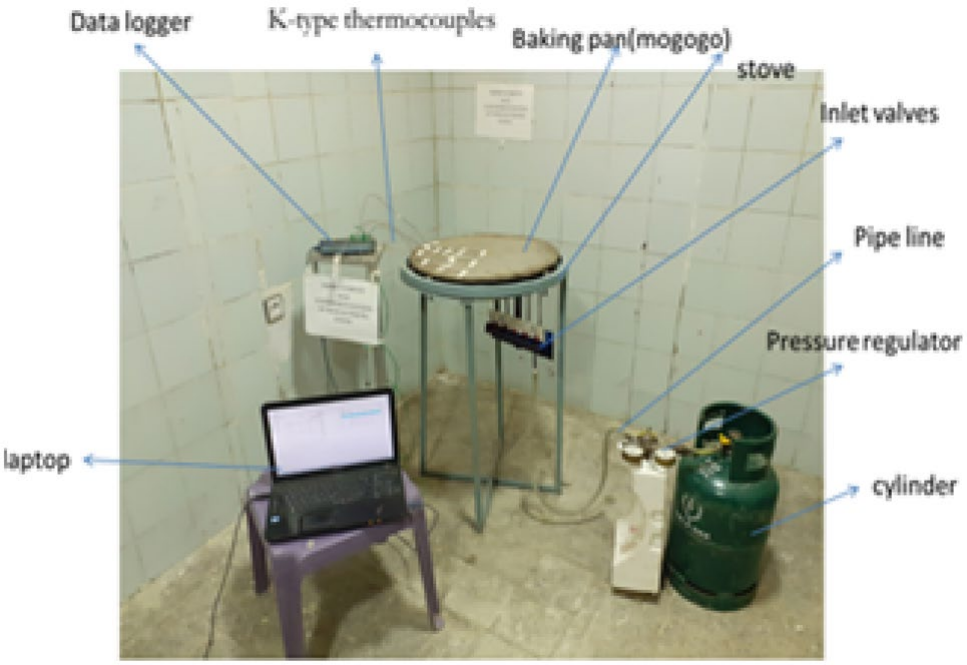
Setup for temperature distribution for all material setup.

**Fig. 18:**
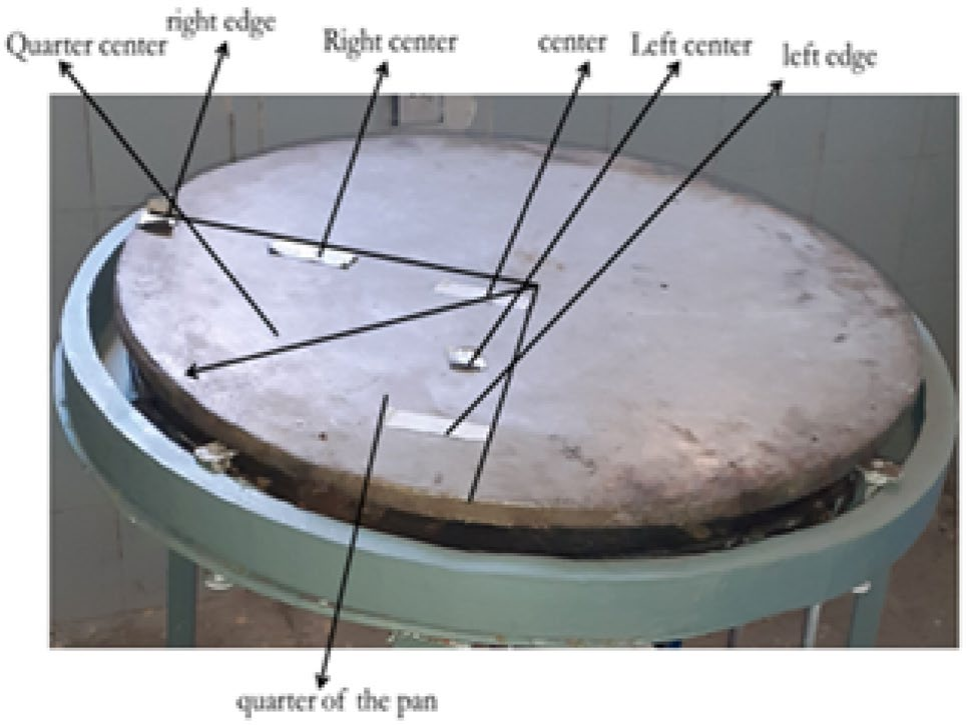
Shown for thermocouple setup on the injera baking pan.

Fifteen kilograms of fermented teff dough were used to conduct baking tests under realistic conditions. Baking time per injera, idle heating time, and total biogas consumption were recorded. 25 guest participants evaluated the resulting injera through a sensory assessment involving texture, appearance, and taste. Thermocouples were also embedded near the baking area to track internal pan temperature during each baking cycle. As illustrated in Fig. 17, the experimental setup for temperature distribution was established for all material configurations to ensure uniform measurement across the stove components. This arrangement allowed for consistent data collection and accurate comparison of temperature profiles under varying material setups. The thermocouple arrangement used for temperature monitoring on the injera baking pan is presented in Fig. 18. This setup enabled precise recording of surface temperature variations during the baking process, ensuring reliable evaluation of the thermal performance of the stove.

## Results and discussion

### Quantitative performance analysis

The performance evaluation of the improved biogas-powered stove was carried out under varying design conditions. The key indicators measured included thermal efficiency, specific fuel consumption (SFC), time to boil, and baking time for Injera. Compared to the traditional three-stone stove, the improved biogas stove demonstrated a significant enhancement in thermal performance. The thermal efficiency increased by 32%, rising from 21% (traditional) to 37% (improved stove^16^. This efficiency is comparable to values reported for optimized household-scale biogas stoves in India and Nepal, which typically range from 35 to 40%^19,20^. Similarly, a study^21^ reported 25% efficiency for a moderately insulated biogas stove, reinforcing that our concentric-loop and variable-hole design provides a clear performance gain. This improvement is attributed to optimized burner geometry, improved insulation, and better combustion control. Specific fuel consumption (SFC) was reduced to 0.48 m^3^/kg, outperforming the 0.62 m^3^/kg reported^22,23^, for fixed-dome stoves in Ethiopia. Comparable reductions in SFC have been documented in advanced designs incorporating swirl inlets and staged combustion^15^, suggesting that better mixing and combustion completeness explain our improved results.

The average flame temperature of 1954 K measured in this study exceeds values reported in other experimental biogas stove assessments, which often remain below 1800 K^3,5^. This improvement is consistent with^4,24^ and who demonstrated that improved mixing and controlled H_2_S concentrations enhance combustion kinetics and raise flame stability in HCCI engine experiments. Similarly, our finding that pressure regulation and variable hole sizing ensure flame uniformity aligns with CFD-based optimization studies of porous burners^25,26^ and earlier injera stove designs^3,20,27^.

Injera baking time in our design (~ 16 min per batch) compares favourably to the ~ 20 min reported^28^ for conventional metal-plate stoves, demonstrating practical benefits of enhanced heat transfer. Comparable time savings were reported^18,23^ in solar steam-based injera baking, though that system faced weather dependency issues.

### Design and engineering interpretation

The improved stove’s concentric burner loops with variable hole diameters directly address the non-uniform flow distribution reported in earlier Ethiopian prototypes^22,28^. Our findings also support insights from^2^ who showed that raw biogas composition strongly affects combustion uniformity and sensitivity, underscoring the importance of adaptive geometric designs for real-world variability. The 21.6% efficiency improvement over the baseline is consistent with global studies of burner optimization, such as^26,29^ who demonstrated that enriched air–fuel mixing significantly improves flame structure and heat release characteristics.

### Socioeconomic and practical impact

In rural Ethiopian households, Injera baking accounts for a large share of daily energy use. The improved biogas stove addresses multiple practical issues, including energy access, fuel cost, safety, and gendered labor burdens^29^. A cost–benefit analysis suggests that a household using this stove can save up to 40% on daily fuel expenses, translating to significant savings over time. In addition, the stove’s fast cooking time allows for better time management, saving approximately 2.5 h per day in combined boiling and baking activities^30^. Such improvements are critical in rural households where daily fuel availability is limited. Similar socioeconomic gains from biogas adoption-fuel savings, health benefits, and time efficiency-have been reported in Uganda^3,8^ and India^5,31^. By delivering culturally appropriate design specifically for injera, this study extends the applicability of those general findings to Ethiopia.

### Burner feeding loop evaluation

Table 2 presents the measured diameters for the first half of the feeding loop during the burner design phase, as illustrated in the corresponding figure. These measurements are critical for assessing flow dynamics and ensuring consistent fuel delivery to the combustion chamber. The data obtained supports nozzle design optimization for achieving uniform pressure distribution and stable flame propagation^27,28^. The stove’s portability, ease of cleaning, and compatibility with locally available materials ensure high usability. Moreover, it contributes to health improvements by significantly reducing smoke exposure, which aligns with SDG 7 (clean energy) and SDG 3 (good health)^30^.

**Table 2:** Summary result of the diameter for the first half of feeding loop five.

Points	Length (m)	Pressure (Pa)	Area in pipe (m^2)	Velocity (m/s)	Diameter (m)
0	1	86.59333422	3.63E−05	0.710951447	0.0017
1	1.05	79.91284916	3.63E−05	0.666516981	0.001755752
2	1.1	72.9278742	3.63E−05	0.622082516	0.001817376
3	1.15	65.73994597	3.63E−05	0.577648051	0.001885981
4	1.2	58.4505411	3.63E−05	0.533213585	0.001962991
5	1.25	51.16113624	3.63E−05	0.48877912	0.002050277
6	1.3	43.973208	3.63E−05	0.444344654	0.002150349
7	1.35	36.98823304	3.63E−05	0.399910189	0.002266667
8	1.4	30.30768798	3.63E−05	0.355475723	0.002404163
9	1.45	24.03304945	3.63E−05	0.311041258	0.002570158
10	1.5	18.26579409	3.63E−05	0.266606793	0.002776088
11	1.55	13.10739854	3.63E−05	0.222172327	0.003041052
12	1.6	8.659339422	3.63E−05	0.177737862	0.034
13	1.65	5.023093376	3.63E−05	0.133303396	0.003925982
14	1.7	2.300137034	3.63E−05	0.088886893	0.004808326
15	1.75	0.591947031	3.63E−05	0.044434465	0.0068
Total		598.0356289	–	–	–

### Existing stove simulation results

A computational fluid dynamics (CFD) simulation was conducted using ANSYS Fluent 15 to assess the performance of the existing biogas-powered injera stove. The analysis focused on understanding the biogas flow dynamics, flame propagation, and heat distribution across the burner’s feeding loops.

### Flow dynamics and velocity distribution

The simulation results revealed that the biogas velocity was highest near the inlet of feeding loop 5, reaching a maximum value of approximately 3 m/s. As the gas progressed through the successive loops (from loop 5 to loop 1), the velocity gradually decreased^28^. This trend is attributed to the cumulative pressure loss and frictional effects along the pipe length as gas escapes through the evenly sized holes^17^. The primary issue observed was the non-uniform velocity distribution across the burner’s holes, despite all holes having identical diameters. This discrepancy led to an unequal volume flow rate of biogas in different regions of the burner, particularly between the outermost and innermost loops.

**Fig. 19:**
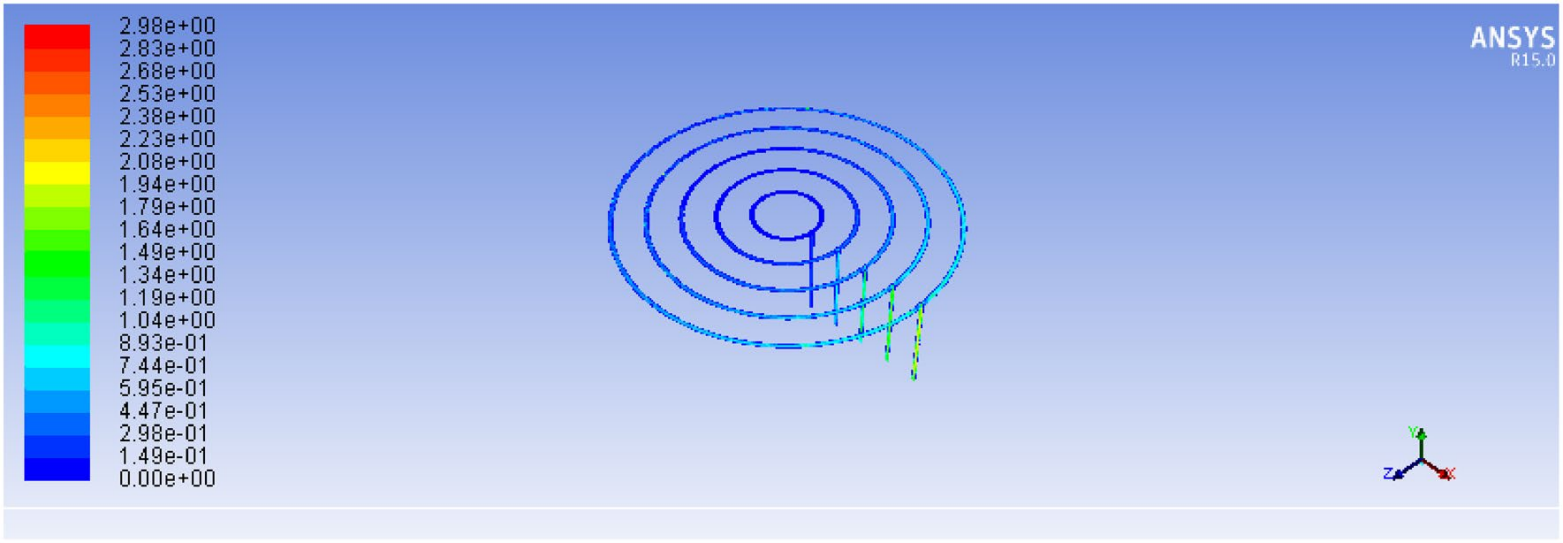
Existing stove velocity simulation result.

Figure 19, below, illustrates the velocity field within the burner geometry, showing clear gradients along the feeding paths. The outermost loops (which receive biogas first) experience stronger flow and higher velocity, while inner loops suffer reduced flow rates^17^. Consequently, holes closer to the gas source exhibit more intense flames, while those farther away produce weaker combustion. As shown in Fig. 19, the ANSYS simulation illustrates the flow distribution pattern across the biogas feeding loops under steady-state operating conditions. The contour plot clearly demonstrates variations in flow intensity along the spiral paths, indicating efficient gas distribution toward the burner outlets.

### Flame characteristics and heat distribution

Due to the velocity imbalance, the flame characteristics (including height, width, and temperature) varied considerably across the burner. In areas with higher velocity, flames were taller and more vigorous, while in areas with lower velocity, flames were shorter and less stable. These differences caused asymmetric heating of the baking surface, which can be problematic for injera preparation, where uniform temperature is crucial.

The CFD model did not include deflection from the baking pan in this version of the simulation, so the flame profile was evaluated based on its natural propagation from the holes. Uneven flame geometry not only reduced thermal efficiency but also contributed to fuel waste and inconsistent baking results.

### Implications for stove performance

The findings demonstrate that the uniform hole diameter in the original design was insufficient to maintain consistent thermal output across the pan surface. Because the biogas flow rate through each hole is a function of both pressure and velocity, the drop in pressure along the pipe directly impacted the heat output in downstream sections.

This non-uniformity is particularly problematic for injera baking, where any inconsistency in pan temperature can result in uneven cooking, poor texture, and undesirable taste.

#### Design shortcomings identified

Key deficiencies in the original design, as revealed through simulation, include:

- Uniform hole diameter despite varying pressure conditions along the feeding loops.
- Multiple sharp turns and bends in the pipeline contribute to additional pressure losses.
- Lack of compensatory mechanisms (e.g., variable hole sizing or staged pressure zones) to maintain even gas distribution. These insights informed the redesign strategy presented in the following sections, emphasizing the need for adaptive geometric modifications and optimized flow dynamics.

#### Improved stove simulation results

**Fig. 20:**
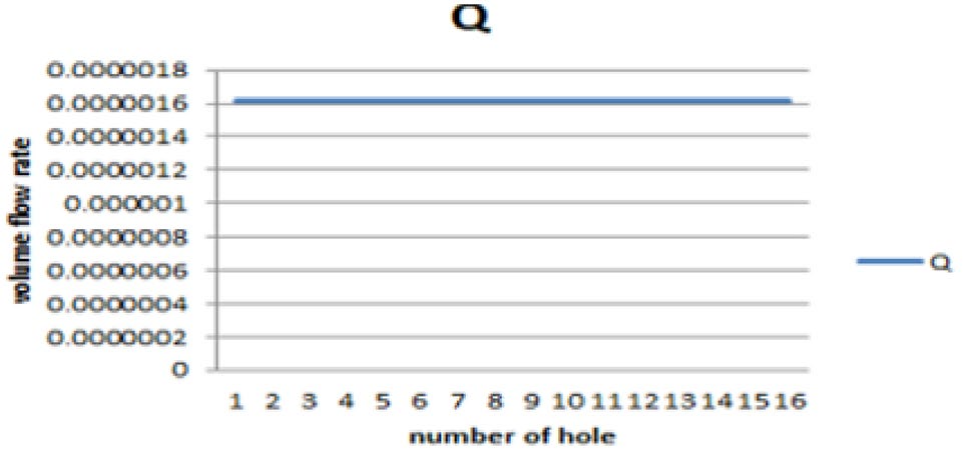
Shown for result of improved stove volume flow rate versus number of holes.

**Fig. 21:**
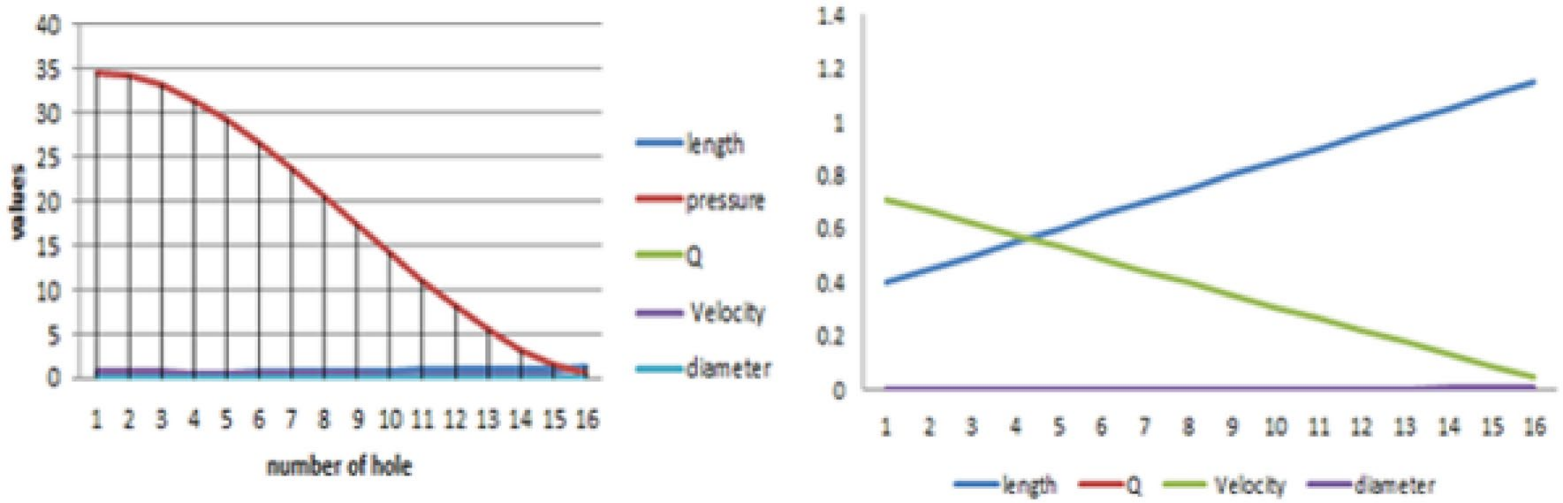
Shown for result of improved stove volume flow rate versus number of holes.

Following the diagnostic assessment of the existing stove design, a newly optimized biogas-powered injera stove was developed and subjected to Computational Fluid Dynamics (CFD) simulation using ANSYS Fluent 15. The objective of the simulation was to evaluate the impact of the improved geometry, specifically the variable hole diameters, optimized pressure regulation, and simplified flow paths, on flame behavior, temperature uniformity, and thermal efficiency. The improved stove simulation result presents the measured diameters for the first half of the feeding loop during the burner design phase, as illustrated below in the corresponding Figs. 20 and 21.

#### Biogas flow and velocity field analysis

**Fig. 22:**
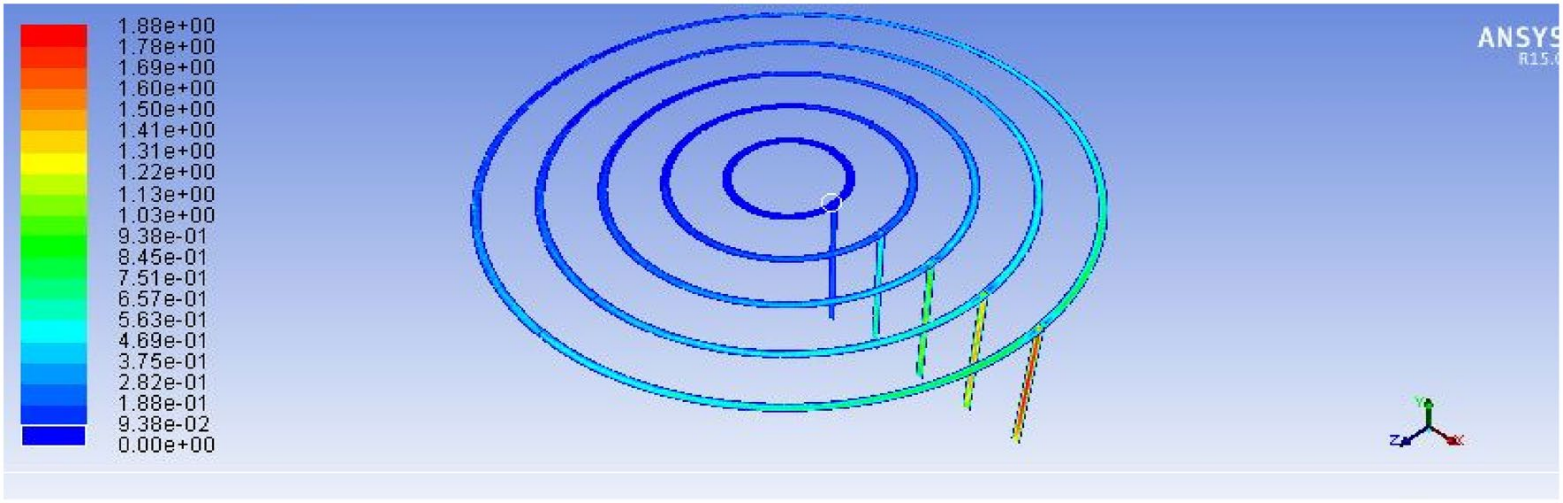
Shown for improved stove velocity simulation results.

The simulation results demonstrated a marked improvement in the flow distribution across the five feeding loops. As shown in Fig. 22: the velocity of the biogas entering the outermost loop (Loop 5) was approximately 1.89 m/s, and it progressively decreased toward the innermost loops in a controlled and predictable manner. Unlike the previous design, where velocity gradients led to uneven gas distribution, the new configuration ensured a relatively uniform volume flow rate across all 93 burner holes.

**Fig. 23:**
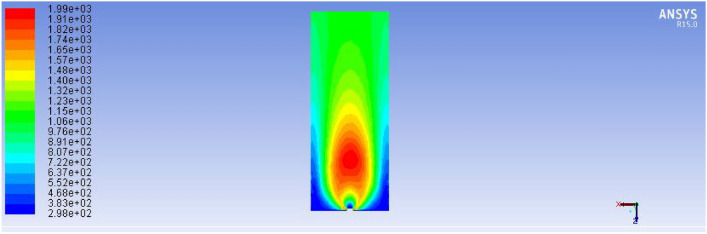
Indicates for improved stove single flame temperature simulation results.

**Fig. 24:**
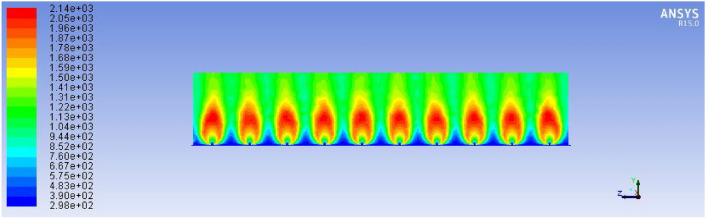
Describes for improved stove side total flame temperature simulation results.

This was achieved by implementing strategically varied hole diameters, smaller near the inlet and larger toward the distal ends, to compensate for the natural pressure drop. Additionally, the new design reduced sharp bends and turns in the piping system, minimizing frictional losses and enhancing laminar flow behaviour throughout the stove structure as shown in Figs. 23 and 24.

#### Flame structure and temperature distribution

The flame characteristics of the new design were significantly more uniform. Simulation of a single flame jet before deflection by the pan surface (Fig. 23) indicated a flame height of 50 mm and a width of 35 mm, with a maximum core temperature of 1954 K. The flame shape was symmetric and stable, which is crucial for efficient heat transfer.

After deflection by the baking pan, the collective flame distribution (Fig. 22) exhibited a consistent flame spread across all holes. Figure 23, shows the total flame temperature field along the pan’s underside, indicating a continuous heating layer, with flames forming a ring-like deflected pattern that reached uniform height and width (average 30 mm × 50 mm). Importantly, all flames touched or nearly touched adjacent flames, minimizing cold spots and ensuring even thermal exposure across the cooking surface. The simulation revealed a dramatic improvement in flame uniformity and heat distribution compared to the earlier prototype. Notably, the new design eliminated the over-heating near the gas inlet and under-heating near the centre, leading to a homogenous thermal field on the baking surface.

#### Thermodynamic and combustion considerations

From a thermodynamic perspective, the new system operated under optimized conditions for non-premixed laminar combustion. The CFD setup used the species transport model with radiation effects and enabled energy conservation. A stoichiometric air-to-fuel ratio was maintained to support complete combustion, as evidenced by the stable blue flame observed in experiments.

**Fig. 25:**
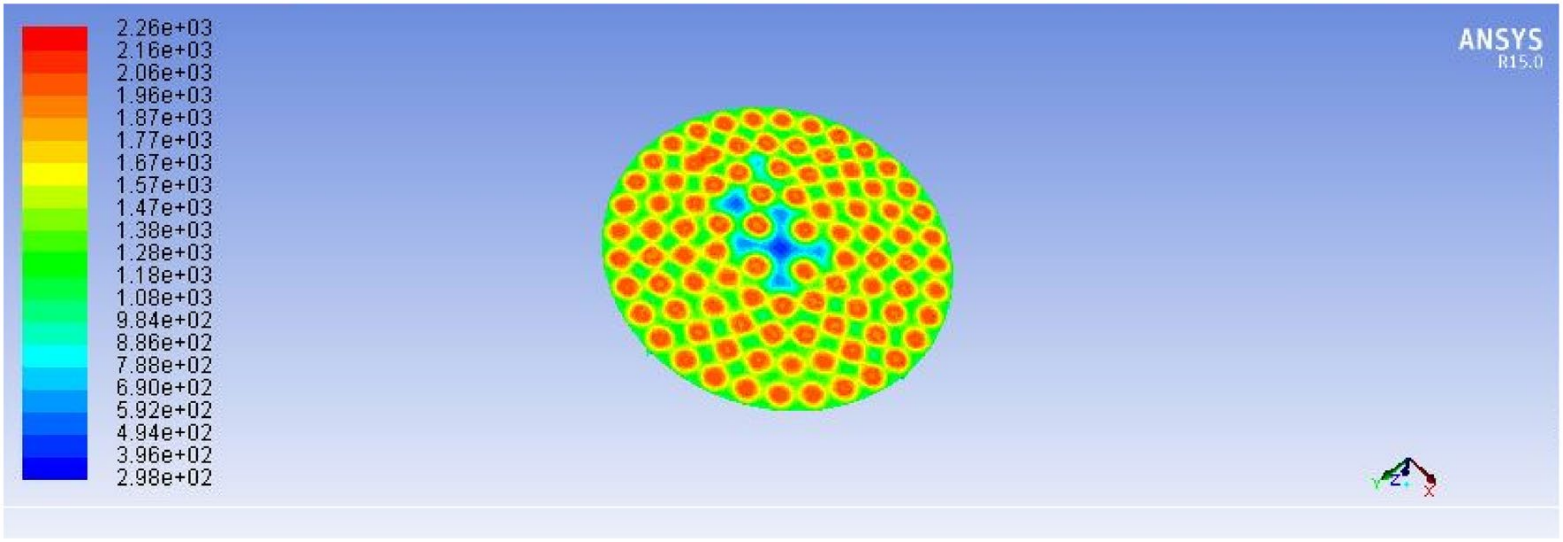
Improved stove simulation results for total flame temperature.

These rates were calculated based on required power (3.3 kW), calorific value of biogas (22 MJ/m^3^), and heat transfer requirements for injera baking. The air inlet rate (1.8033 × 10⁻^3^ m^3^/s) was selected to ensure complete combustion without excessive dilution or heat loss. Figure 25 below describes the improved stove design demonstrates a significant increase in total flame temperature, as predicted by ANSYS R15.0 fluent simulations, indicating enhanced thermal performance and combustion efficiency.

**Fig. 26:**
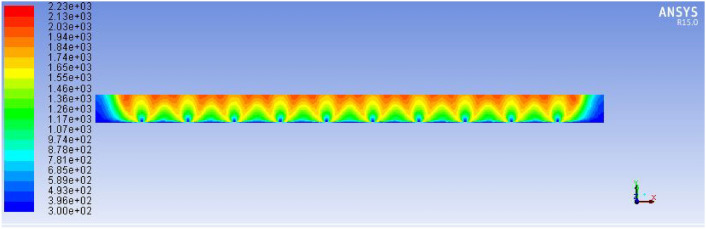
Improved stove simulation results for side view of total temperature.

The temperature distribution of the improved biogas stove was analyzed using ANSYS R15.0 simulation to evaluate heat transfer uniformity and combustion performance. As shown in Fig. 26, the side-view temperature contour illustrates periodic high-temperature zones within the combustion chamber, ranging between approximately 300 K and 2230 K. The highest temperature regions (depicted in red) occur near the burner inlets, where combustion intensity and convective mixing are strongest. The temperature gradually decreases toward the exhaust zone, indicating effective heat dissipation along the stove wall.

This result confirms that the modified design achieves better temperature uniformity and higher flame propagation efficiency compared to conventional stove geometries, which typically suffer from localized hot spots and incomplete combustion.

#### Key improvements over the existing stove

Compared to the original design, the simulation of the improved stove revealed several critical performance enhancements:

- *Uniform biogas distribution* Achieved through adaptive hole sizing and balanced loop geometry.
- *Improved flame symmetry* Ensuring predictable heat delivery across the entire pan.
- *Optimized thermal contact* With deflected flames forming a continuous thermal layer beneath the pan.
- *Lower pressure requirements* The total operating pressure was reduced from 25,000 Pa to 2148 Pa, conserving biogas while maintaining performance.
- *Higher predicted thermal efficiency* Confirmed experimentally in Section “Injera baking performance”.

This section illustrates how strategic design modifications, informed by simulation, are directly translated into better fuel economy, thermal stability, and suitability for injera baking. Let me know if you’d like a side-by-side comparative figure or table for this section.

### Experimental validation

To validate the numerical simulation outcomes and assess the practical performance of the improved biogas-powered injera stove, a series of carefully structured experimental tests was conducted. These tests focused on flame propagation, temperature distribution, biogas consumption, and overall baking performance. All experiments were carried out at Mekelle University in September and October 2024 under controlled laboratory conditions.

#### Flame characteristics and combustion behaviour

A critical first step was to assess flame behaviour under real operating conditions. The flame was generated by burning compressed biogas, regulated at 2152 Pa and supplied through the five-loop burner system. The flame testing confirmed the simulation results. Visual observations and video recordings using a Samsung Galaxy A10 camera showed a consistently blue flame across all holes, indicative of complete combustion and minimal carbon monoxide production. The flames were stable and symmetrical with minimal flickering, indicating sufficient mixing of biogas and air. Measured flame dimensions were:

- Height: 33 mm
- Width: 46 mm

**Fig. 27:**
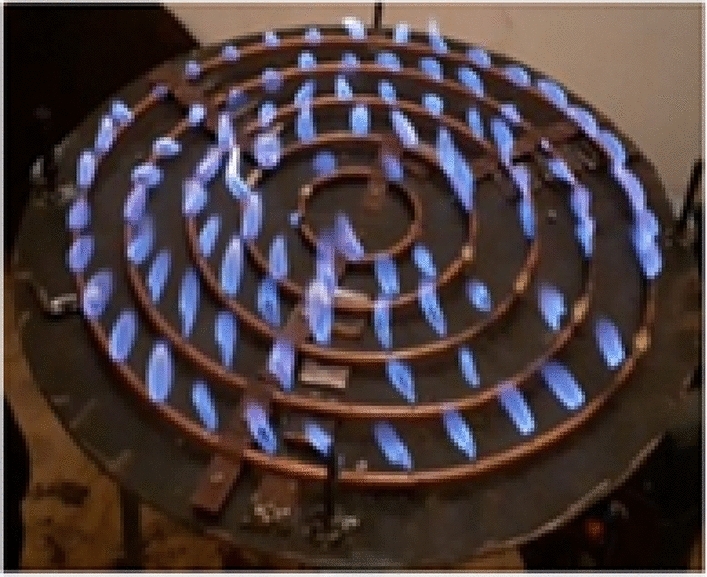
Flame propagation and characteristics indicates for flame distributions in holes.

**Fig. 28:**
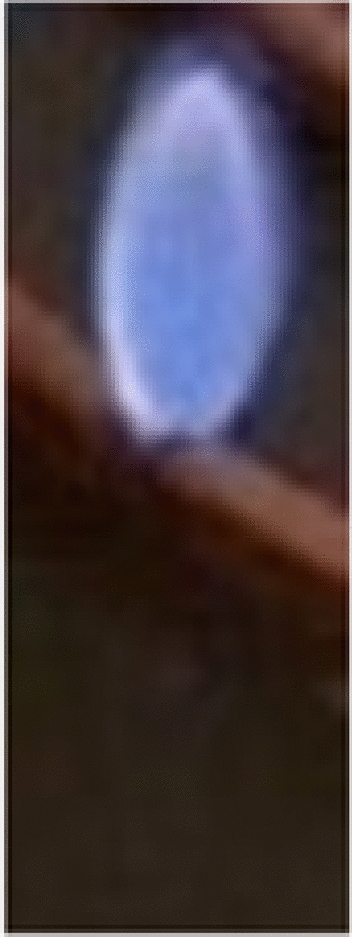
Flame propagation and characteristics single flame.

**Fig. 29:**
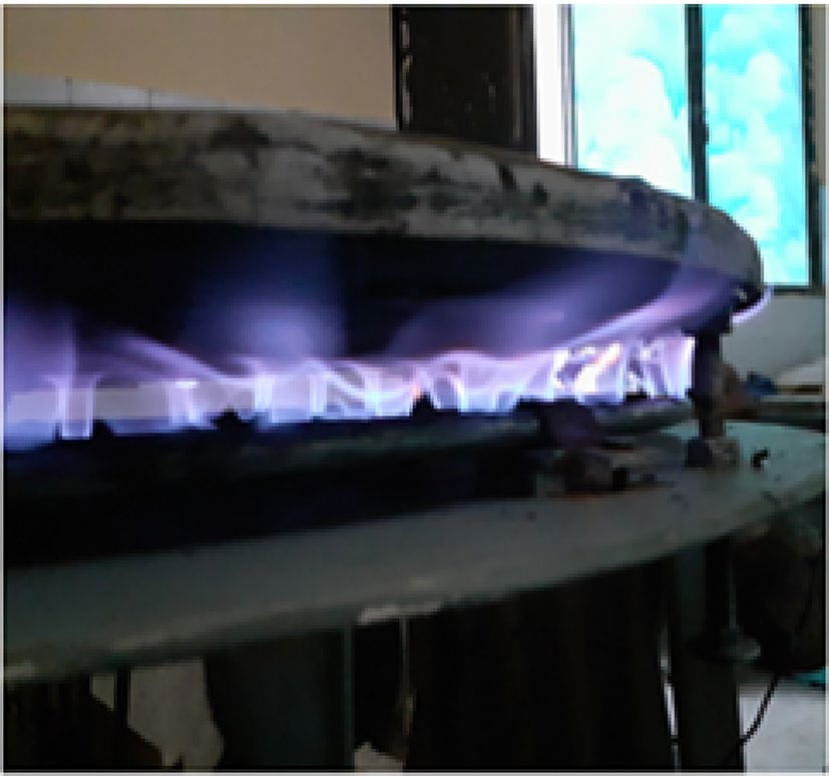
Flame propagation and characteristics for flame distribution deflected by baking pan.

These values closely matched the simulation predictions and demonstrated uniform combustion across all burner loop). The successful control of flame parameters was a direct result of the redesigned burner geometry, variable hole diameters, and improved flow regulation, as shown below in Figs. 27, 28 and 29.

#### Temperature distribution on the baking pan

**Fig. 30:**
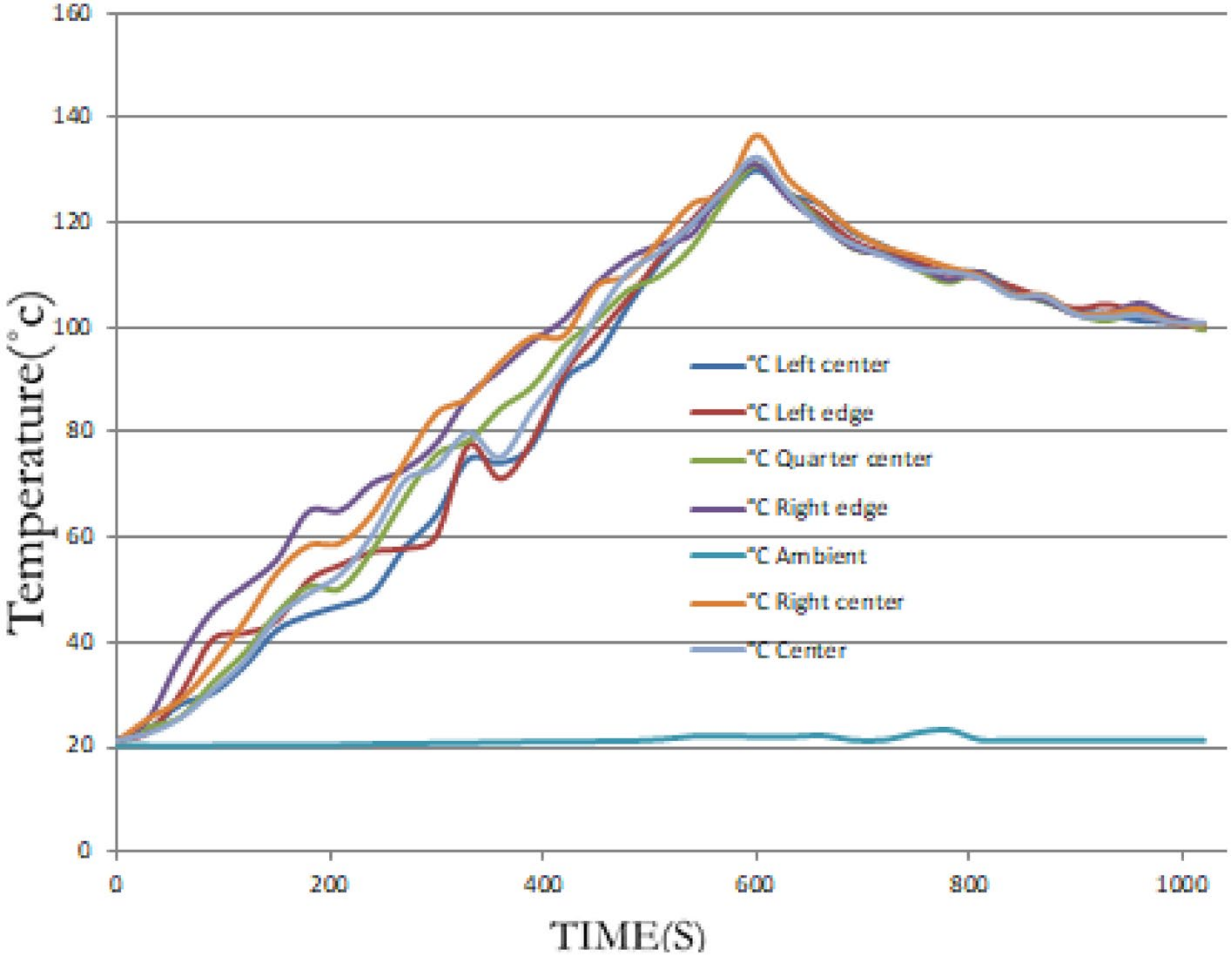
Temperature distributions on the pan during heating and cooling.

The uniformity of heat transfer was evaluated by measuring the temperature distribution across the aluminium baking pan with K-type thermocouples. Six thermocouples were carefully positioned in a quarter section of the pan to detect temperature differences along the x- and y-axes described in Fig. 30. The stove was operated at maximum power, and temperature data were recorded every minute.

The results, shown in Fig. 30, demonstrated smooth and uniform temperature rise across all zones of the pan. Starting from an ambient temperature of 21 °C, the central region of the pan reached 135 °C in just under 10 min. Other positions recorded the following temperatures at 9 min and 50 s, which is enough for baking ^18^. The maximum temperature difference across the pan was less than 10 °C, which is well within acceptable limits for consistent injera baking. The uniform thermal field is attributed to the effective deflection and overlapping of flame jets beneath the pan, as observed in the simulation.

After baking, natural cooling occurred via convection, and the pan temperature dropped to approximately 32°Cover two hours. This gradual cooling confirms the effectiveness of the fiberglass insulation in minimizing heat loss from the stove’s sides.

#### Injera baking performance

A full-scale injera baking trial was conducted using 15 kg of fermented dough, yielding approximately 25 injera. The baking process was closely monitored to evaluate operational efficiency, fuel consumption, baking time, and product quality.

- Time to reach baking temperature (140 °C): 10 min
- Baking duration per injera: 2 min
- Idle reheating time between cycles: 1 min
- Total biogas consumed: 1.3 m^3^

**Fig. 31:**
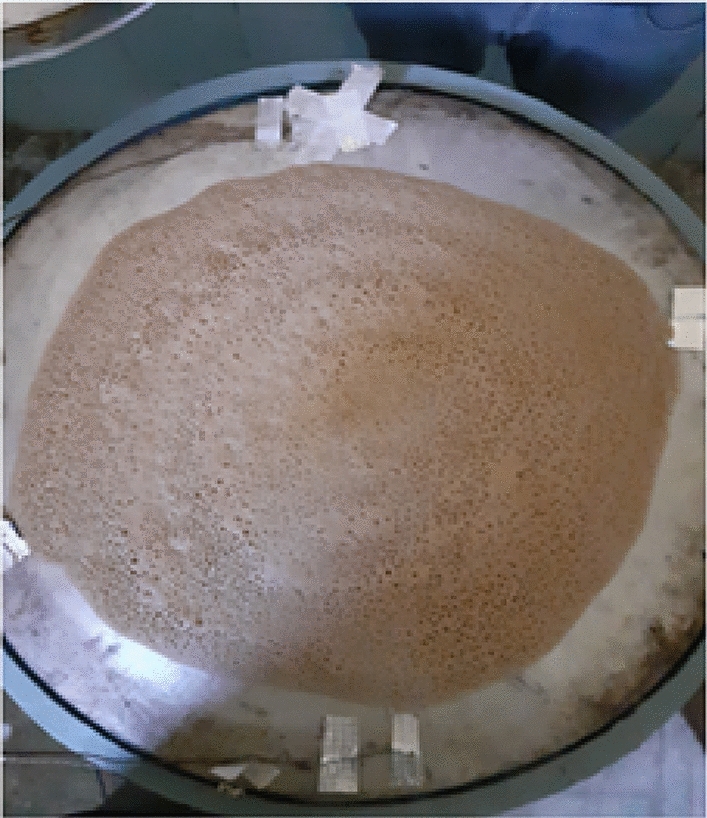
Shown for baking injera test, injera on baking pan.

**Fig. 32:**
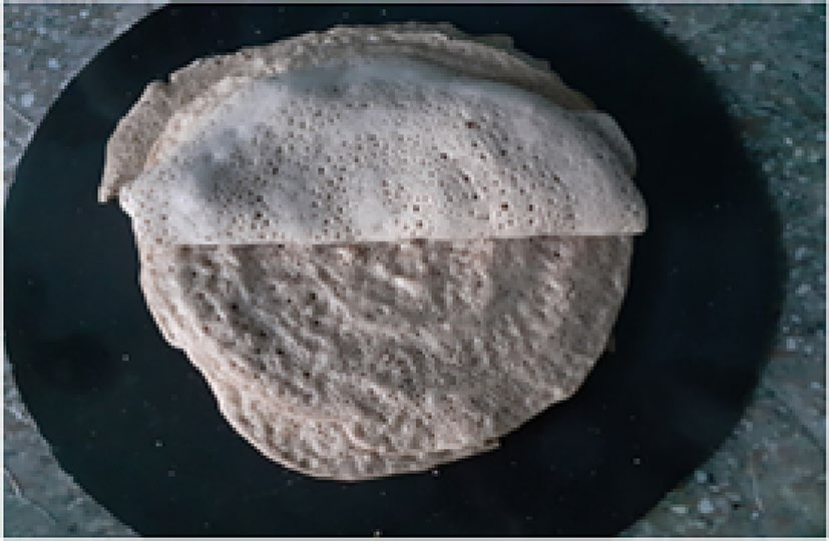
Indicates for baked injera.

The injera produced during the trial was visually uniform and well-cooked (Figs. 31 and 32). The bottom and top textures were consistent, and the typical spongy structure was retained. Figure 25c shows the baking timeline, where minimal downtime and continuous baking operations were maintained, confirming the stove’s suitability for household-scale applications.

**Fig. 33:**
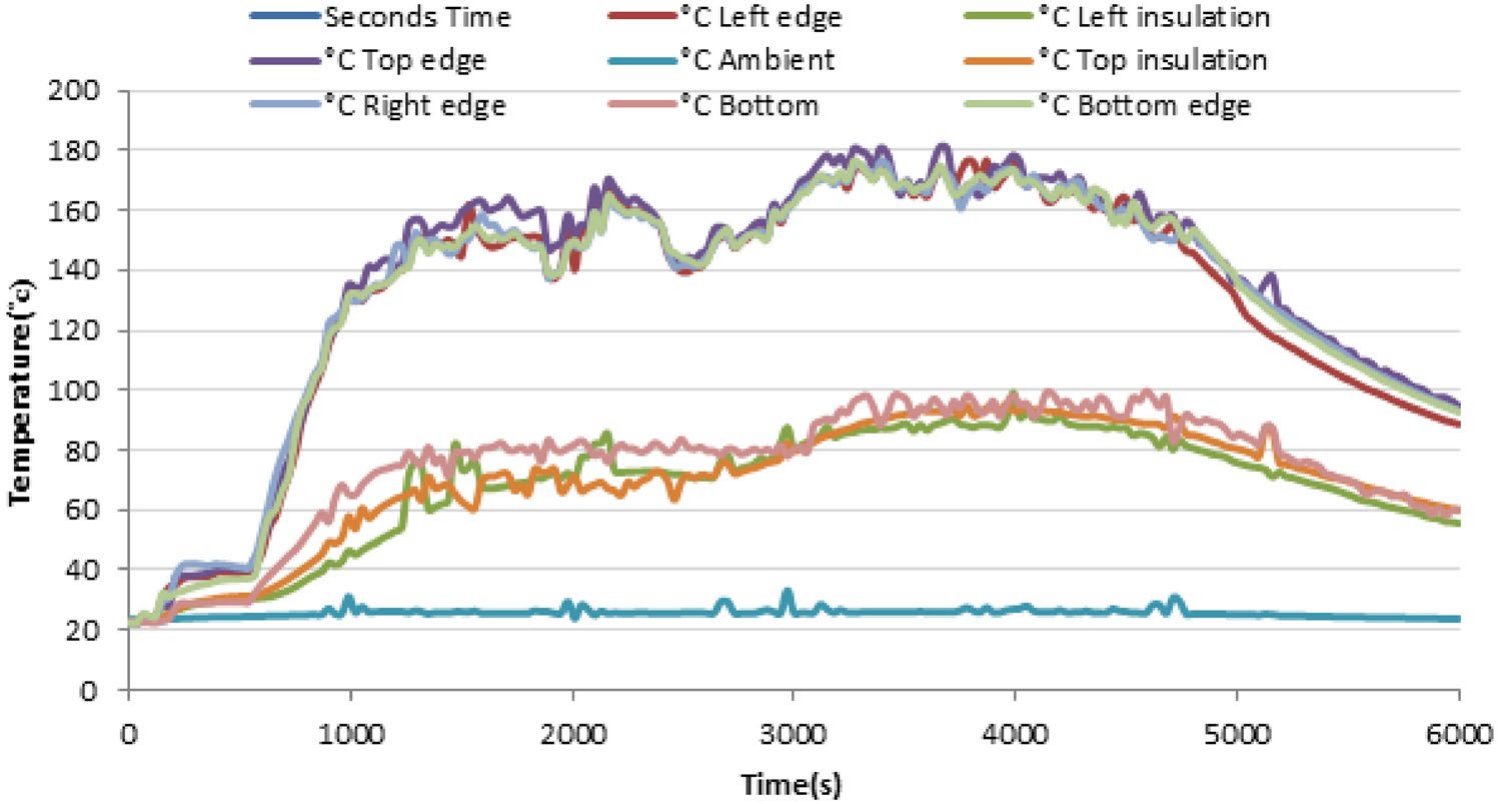
Shown for baking injera test, graph of baking injera.

To validate the simulation results, experimental temperature measurements were conducted during the injera baking test of the improved stove design. As shown in Fig. 33, the temperature variation was recorded at multiple locations of the stove including the top, bottom, left, right, edge, and insulation layers over a baking cycle of approximately 6000 s. The results show a rapid temperature rise during the initial heating phase, followed by a steady-state range of 140–170 °C across the upper and edge regions of the baking surface. This temperature profile corresponds to the optimal range required for uniform injera baking and moisture evaporation.

This Fig. 33 provides experimental validation of the stove’s temperature performance during injera baking, showing temporal temperature variation at different locations of the baking surface and insulation.

The left and top edges exhibited slightly higher temperature gradients compared to the bottom and right sides, which can be attributed to asymmetric heat transfer and localized air convection inside the combustion chamber. The insulation layers maintained a moderate temperature level between 60 °C and 90 °C, confirming effective thermal retention and minimized heat loss to the surrounding environment. These results agree well with the numerical simulation in Fig. 26, indicating that the redesigned stove geometry achieves both high combustion efficiency and uniform thermal distribution along the baking surface.

#### Fuel efficiency and operational parameters

Biogas consumption was measured using a calibrated flow meter. The average biogas consumption rate was 0.9 m^3^ per baking cycle, corresponding to 43.11% thermal efficiency, an increase from the 35.44% observed in the previous stove design as indicated in Table 3. This efficiency improvement is linked to:

- Better combustion due to air-biogas mixing
- Reduced heat loss through insulation
- Optimized flame contact with the pan
- Uniform flame distribution and reduced energy wastage

**Table 3:** Summary of experimental findings.

Parameter	Existing stove	Improved stove	Improvement (%)
Flame uniformity	Uneven	Uniform	Qualitative improvement
Average flame temperature (K)	~ 1700	1954	+ 14.9%
Time to reach 140 °C	> 12 min	< 10 min	− 16.7%
Biogas consumption per batch	1.3–1.5 m^3^	0.9 m^3^	− 30%
Thermal efficiency (%)	35.44	43.11	+ 21.6%
Weight of stove (kg)	25	22	− 12%
Sensory acceptability	Not tested	High	Qualitative improvement

Additionally, the redesigned stove weighed 22 kg, down from 25 kg in the original version, enhancing portability and ease of installation for rural households.

#### Sensory evaluation of baked Injera

To evaluate the acceptability of the biogas-baked injera, a sensory evaluation was conducted with 25 volunteer participants. Each guest assessed three key attributes:

- Visual appeal
- Taste
- Size and texture consistency

**Fig. 34:**
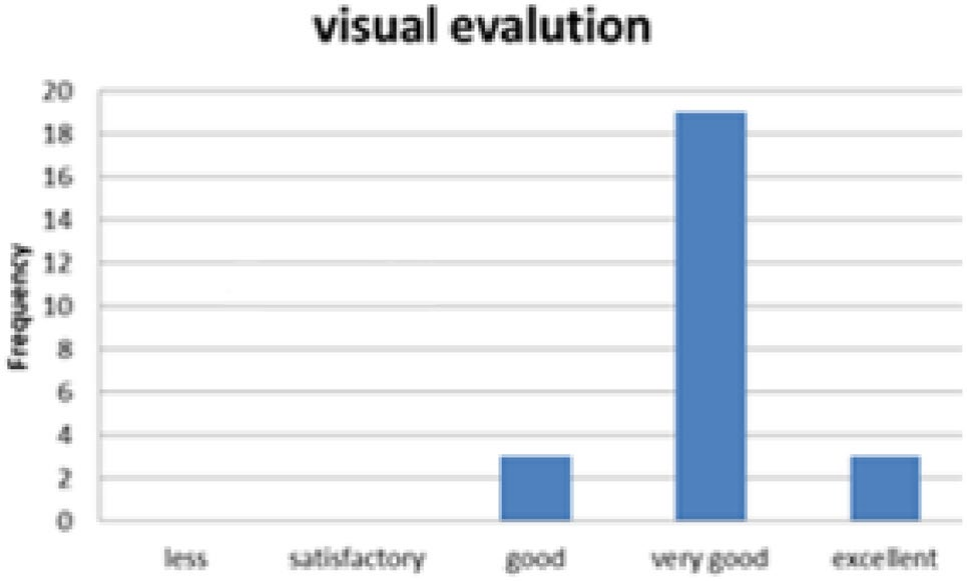
Shown for sensory visual evaluation results.

**Fig. 35:**
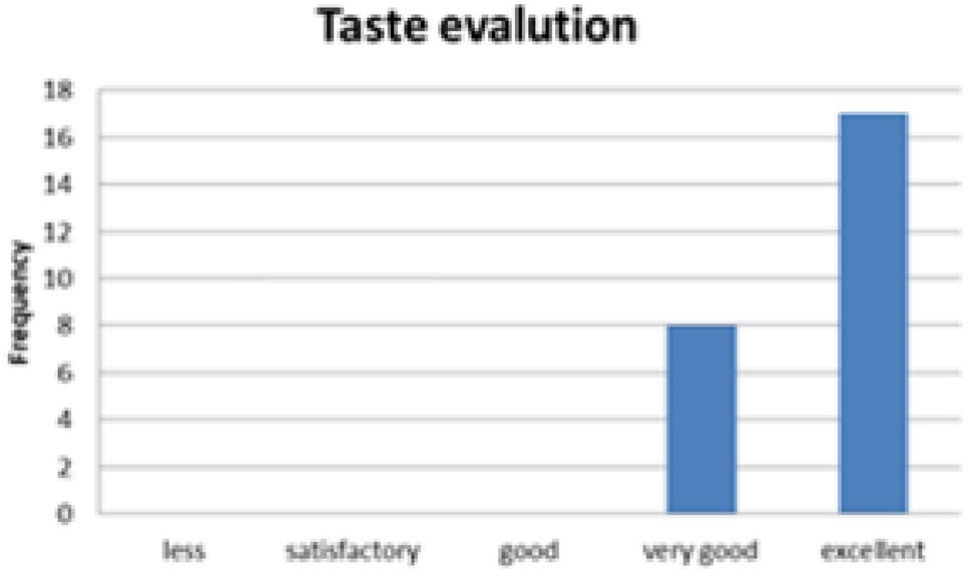
Indicated for sensory test evaluation results.

**Fig. 36:**
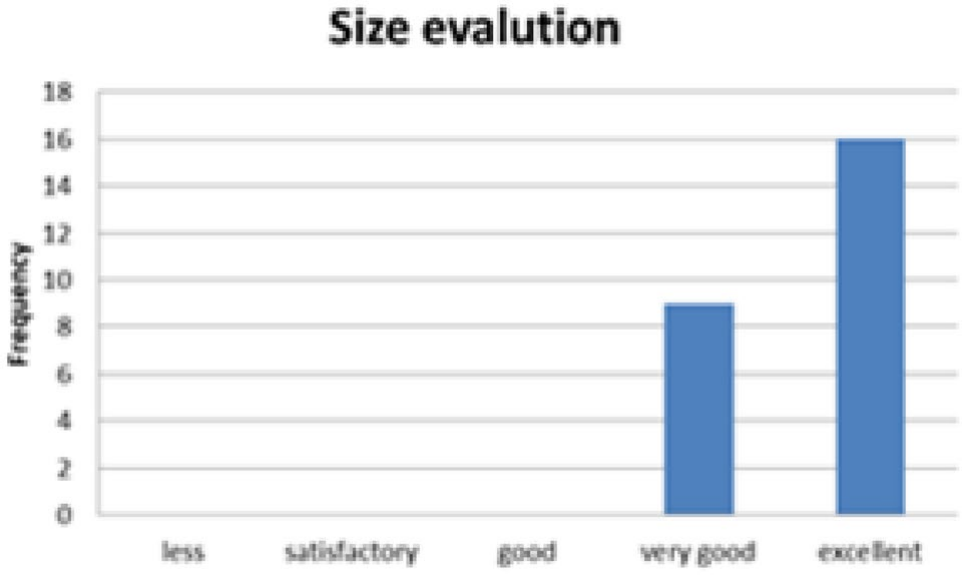
Illustrated for sensory size evaluation results.

As illustrated in Figs. 34, 35 and 36, all participants rated the injera highly across all parameters. Cumulative ratings exceeded the midline threshold across the board, with most scores falling in the upper quartile. This confirms that biogas does not adversely affect the sensory quality of injera, making it an acceptable alternative to traditional firewood or electric baking methods.

## Conclusions and limitations

### Conclusions

This study presents the design, simulation, fabrication, and experimental validation of an improved biogas-powered injera baking stove tailored for rural Ethiopian households. Table 3 presents the experimental results, showing a significant improvement in thermal efficiency from 35.44% for the existing stove to 43.11% for the improved model. The primary goal was to address critical challenges associated with traditional and earlier biogas stove designs, namely, inefficient fuel usage, uneven heat distribution across the baking surface, and insufficient thermal performance for consistent injera preparation.

Through the use of advanced Computational Fluid Dynamics (CFD) modelling, the redesigned stove integrated key performance enhancements, including optimized biogas flow paths, variable hole diameters, and concentric loop geometry. Simulation results revealed that the new burner design achieved a uniform biogas distribution, consistent flame propagation, and a maximum flame temperature of 1954 K. Unlike the conventional stove, which suffered from localized overheating and poor flame control, the improved design provided balanced thermal coverage beneath the pan, critical for producing high-quality injera.

Experimental results strongly validated the simulation outcomes. The stove successfully achieved the required baking temperature of 140 °C in under 10 min, with minimal variation across different regions of the pan. Uniform flame height and width were observed across all burner loops, confirming the effectiveness of the pressure-balancing and hole-sizing strategy. Furthermore, the stove demonstrated a significant reduction in biogas consumption, averaging 0.9 m^3^ per full baking cycle, and achieved a thermal efficiency of 43.11%, an increase of approximately 21.6% compared to the previous model. This makes it more compatible with the gas output of small-scale household biogas digesters commonly used in rural Ethiopia.

Importantly, the sensory evaluation of the biogas-baked injera, conducted with 25 participants, confirmed that the quality of the final product was equivalent in taste, texture, and appearance to that produced using traditional biomass or electric stoves. This finding highlights the potential for wide acceptance of the technology among end users, provided the stove is made available at an affordable cost and accompanied by user training.

In addition to performance gains, the improved stove offers practical advantages. Its weight was reduced from 25 to 22 kg, enhancing portability and ease of installation. The inclusion of 20 mm fiberglass insulation also contributed to reduced heat loss and improved safety for users, especially in enclosed cooking environments.

Overall, this research demonstrates the technical feasibility and practical viability of a biogas-based solution for injera baking. It offers a clean, efficient, and culturally appropriate alternative to biomass-based cooking methods, thereby contributing to Ethiopia’s broader goals of reducing deforestation, improving household air quality, and promoting sustainable rural energy systems. Future work may explore further integration of automatic control systems for flame regulation, adaptation to other traditional food applications, and wider community-based deployment strategies.

### Limitations of the study

While the outcomes of this study demonstrate substantial improvements in the design and performance of biogas-powered injera baking stoves, several limitations must be acknowledged that may affect the generalizability and scalability of the findings.

First, the experimental validation was conducted under controlled laboratory conditions using compressed biogas with regulated pressure and flow. Although this approach ensured consistency and accuracy in performance testing, it may not fully replicate real-world rural environments where biogas pressure and quality can vary significantly depending on digester type, feedstock composition, ambient temperature, and user handling practices. As such, the stove’s performance may differ under fluctuating field conditions.

Second, the study focused primarily on thermal efficiency, temperature distribution, and flame characteristics without an in-depth evaluation of long-term durability, user ergonomics, or safety over extended use. The durability of materials, especially under frequent heating cycles and exposure to moisture, was not examined through accelerated aging or stress testing. Similarly, while the stove includes insulation to minimize heat loss, a detailed assessment of heat radiation to the user and the surrounding space was beyond the scope of this study.

Third, the economic feasibility and social acceptability aspects were not comprehensively addressed. While the sensory evaluation confirmed the quality of biogas-baked injera, other adoption factors, such as cost of stove fabrication, maintenance requirements, availability of spare parts, and cultural perceptions of biogas use for cooking, were not systematically studied. This may affect user uptake and the long-term sustainability of the technology in rural communities.

Furthermore, the CFD simulation assumed ideal flow and combustion conditions, including laminar flow, steady-state operation, and homogeneous biogas-air mixing. Real-world operation may involve transient behaviour, partial combustion, or local turbulence, which were not modelled in this work. These simplifications could introduce discrepancies between simulated and actual flame behaviour.

Finally, the stove design was tailored specifically for injera baking using a fixed pan size and burner configuration. Its adaptability to other cooking applications or pan geometries remains untested. Customization for households with different cooking needs or energy availability may require additional design modifications and validation. Addressing these limitations in future research through field trials, user feedback, economic analysis, and extended performance testing will be crucial to ensure the wide-scale adoption and long-term success of biogas-powered injera stoves in Ethiopia and similar contexts.

## Supplementary Information

Supplementary Information.

## Data Availability

The datasets are available from the corresponding author upon reasonable request.
