# Supplementary material for: Design optimization and experimental validation of a biogas-powered stove for energy efficiency in injera baking in rural Ethiopia
Source: Sci Rep. 2025 Nov 24;15:41479. doi: 10.1038/s41598-025-25412-7 (PMC12644874; doi:10.1038/s41598-025-25412-7)
Supplement: Supplementary file 1 — Supplementary Information. [file 41598_2025_25412_MOESM1_ESM.docx]

**Appendices:**

**This appendix consists three sub appendixes, appendix, appendix, and appendix.**

**Appendices A:** **Contains some common abbreviations used throughout the paper**

| **Abbreviations** | **Meaning** |
| --- | --- |
| LPG | Liquid Pressurized Gas |
| NBPE | National Biogas Program of Ethiopia |
| FVE | Finite Volume Element |
| CFD | Computational Fluid Dynamics |

**Appendices B:** **Contains some common** list of acronyms and abbreviations

**used throughout the paper**

CH4 Methane

CO2 Carbon Dioxide

CO Carbon monoxide

H2S Hydrogen sulfide

LPG Liquid Pressurized Gas

H2 Hydrogen

NBPE National Biogas Program of Ethiopia

CFD Computational Fluid Dynamics

Sl Vertical Velocity

Cp Specific Heat

J Joule

FDS Fire Dynamics Simulation

HRR Heat Release Rate

MPF Multiple Pool Fire

FVM Finite Volume Element

Vz Velocity in Z Direction

Vθ Velocity in $\theta$ Direction

Vr Velocity in r Direction

P Pressure

$\Delta P$ Change in Pressure

ρ Density

C1 Constant

R Radius

A Area

μ kinematic viscosity

Q Flow rate

P Power

E Energy

T time

Cv Specific Heat

Qt Total Volume Flow Rate

Ap Area of Pipe

Vh Velocity of Hole

Ah Area of Hole

Dh Diameter of Hole

K Kelvin

Kpa Kilo Pascal

**Appendices C:** **Graphical Abstract of the paper**


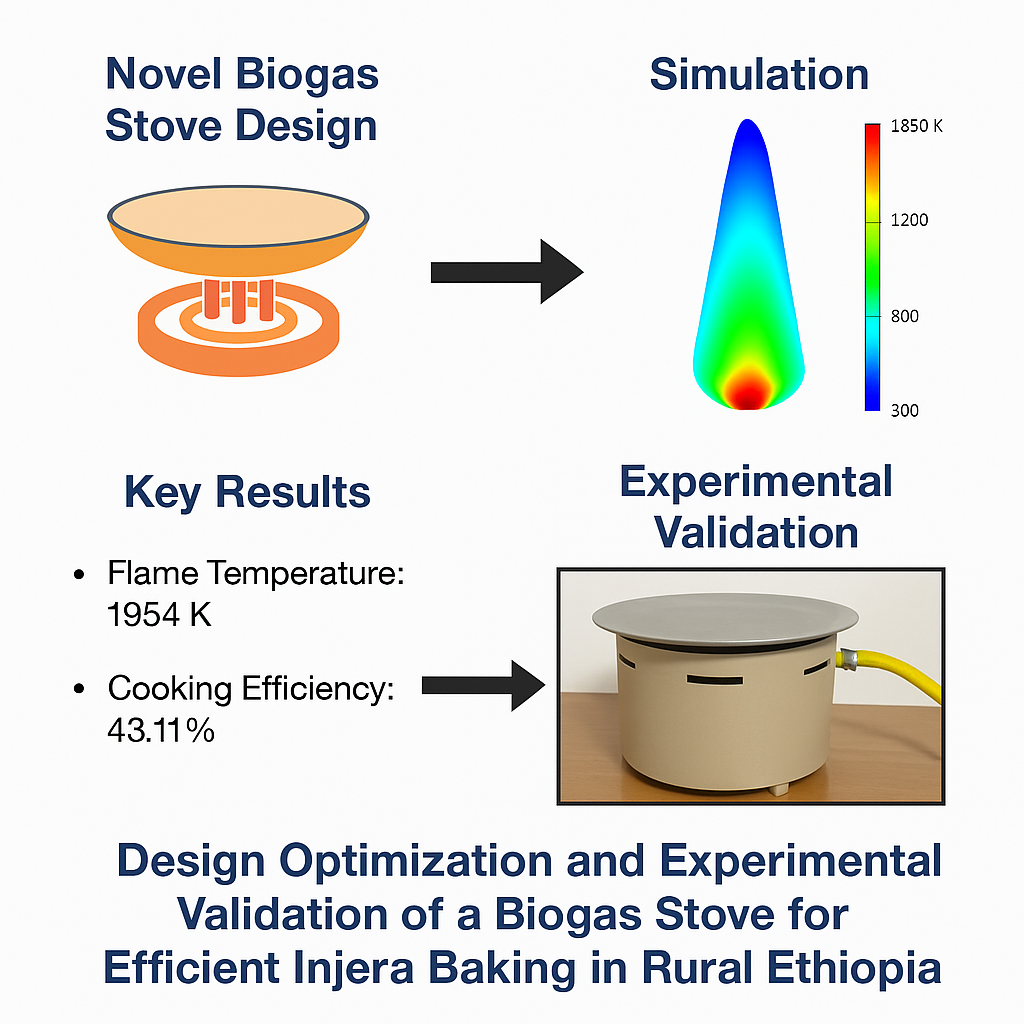


Figure 1: Graphical abstract

**Appendices D:** **Model of** biogas flows in a pipe


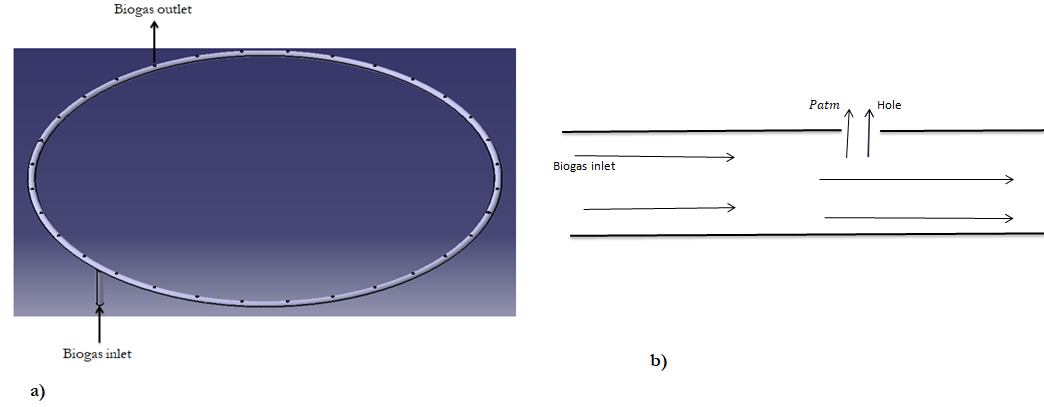


Figure 2: A) 3D pipe draw in CATIA b) biogas flows in a pipe

**Appendices E:** **Flame simulation of biogas**


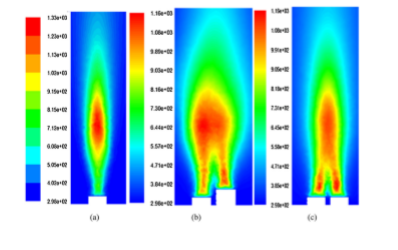


Figure 3: Biogas flame simulation in FDS
